# The importance of structure: Using targeted rewiring to explore social networks property interdependencies

**DOI:** 10.1371/journal.pone.0336496

**Published:** 2026-03-20

**Authors:** Cristina Chueca Del Cerro, Jennifer Badham

**Affiliations:** Department of Sociology, Durham University, Durham, United Kingdom; Hiroshima University: Hiroshima Daigaku, JAPAN

## Abstract

Social networks typically have skewed degree distributions and relatively high clustering and assortativity coefficients. Some studies have explored the relationships between these properties, but have given limited attention to social networks and have found conflicting evidence. To expand our understanding of the ways that properties constrain each other in social networks we use separate degree-preserving rewiring algorithms to manipulate assortativity, clustering coefficient and mean geodesic of networks constructed from seven diverse empirical degree sequences. We measured centrality (mean and Gini coefficient of several measures), clustering, assortativity and network distances. Only a small number of property pairs showed a relationship. Further, where interdependencies do exist, they are conditional and occur only for specific value ranges or a subset of the tested networks.

## 1 Introduction

Social networks exhibit specific structural features that make them different from other networks. Among the common characteristics of social networks are a skewed degree distribution, where a few nodes have many connections while most have few [1]. Those connections tend to have distinctive patterns: (degree) assortativity, where well-connected nodes disproportionally connect with other well-connected nodes [1,2]; and a high clustering coefficient, forming tight-knit groups or communities [3]. Despite these communities, path lengths are short [4], facilitating quick information transfer and network navigability. These features of social networks profoundly shape how information spreads, communities form, and interactions unfold. Understanding these structural properties and the relationships between them is crucial to unravel the complexities of social systems.

Previous explorations of these relationships have manipulated properties through edge rewiring, often involving edge swapping. Holme and Zhao [5] varied both assortativity and clustering coefficient to sweep the mutual property space. The rewiring started from four biological network degree sequences. They found mostly a positive relationship between assortativity, clustering coefficient and the average path lengths. The exception to this was an increase in both assortativity and clustering coefficient associated with decreased mean geodesic for the rewired gene network. Similarly, Xulvi-Brunet and Sokolov [6] found positive associations while manipulating only assortativity on synthetic Barabasi-Albert networks [7].

Alstott and colleagues [8] explored the potential of a rewiring mechanism to quickly increase clustering coefficient. They used two synthetic networks and two empirical Facebook college friendship networks [9]. Most of their results focussed on the algorithm performance. However, they found that average path length increased with the clustering coefficient, slowly at first but with a much greater impact at higher values of clustering coefficient.

Foster et al. [10] manipulated assortativity, homophily, and clustering, and also measured community structures using modularity. They used the degree sequences from three empirical networks and an [11] generated one. They found an asymmetric association; across all four networks, increasing clustering coefficient always increased assortativity but the reverse was not true. When manipulating assortativity, the two properties were only associated for those degree sequences with a broad degree distribution (high degree inequality). They also proffer an explanation; where there are only a small number of high degree nodes, constraining to high assortativity requires those nodes to connect, which in turn creates many triangles as they are likely to have many common neighbours. Modularity was also strongly associated with clustering, but only slightly associated with assortativity.

Looking instead at the structural importance of nodes in networks, Schoch and Shafi [12] examined the relationship between the order of centrality indices values and topological properties of networks (including assortativity and clustering coefficient). There were only weak relationships between the topological structures and the centrality measure ranks in their set of 1,163 empirical networks. However, they found that three centrality measures (degree, closeness, eigenvector) were relatively consistent in their ranks across the property space while betweenness displayed different behaviour.

Communication and infrastructure networks have additional important structural properties, such as resilience in the face of random or targeted node removal [13,14]. Optimisation algorithms that involve edge swapping have also been used to examine the relationship between these properties, particularly how to design networks that are robust against different types of attacks simultaneously [15]. As social networks arise from social interactions rather than being externally designed, these properties are not features of social networks.

We extend this line of research in two ways. Social networks are different from other networks [1] and they have received limited attention in the literature. Previous research (see [5,8,10]) has focused on other types of networks, pseudo-social, non-social, and synthetic, of various sizes (N=100-19,680) and edge densities (0.000 to 0.051). Here instead we focus on small-size social networks (N=101-610, density=0.006-0.148) with diverse social relationships. We use the degree sequences of these empirical networks to explore the interdependencies of their structural properties. Secondly, previous research has predominantly focused on a narrow subset of structural properties that are frequently observed across networks. Here, we expand the structural properties that we measure to understand these relationships more systematically.

We start by discussing the structural properties of our seven empirical social networks before describing the three rewiring algorithms we have designed. Afterwards, we present our experimental design and analyses of the structural property interdependencies. We end with a discussion about the relationships between these properties and the extent to which structural features of social networks can be independently manipulated. Our interest is practical, we wish to generate artificial social networks with specific property values and therefore need to understand potential constraints and whether some properties are easier or harder to control independently.

## 2 Methodology

Our general approach is to extract degree sequences from empirical social networks and use those to construct random starting networks using the Configuration model [16]. We rewire those starting networks in separate experiments to increase assortativity, increase clustering coefficient, or decrease mean geodesic. During the rewiring, we periodically measure a range of properties. Where the properties are calculated for each node or pair of nodes, both the average value and the Gini coefficient of the distributions are reported. The Gini coefficient is a measure of variation that deals well with non-normal distributions [17].

Measures of network centrality reflect the relative importance of nodes in a network. Degree is the number of edges attached to a node [18]. Betweenness measures how often a node appears on the shortest path between other pairs of nodes [19]. Closeness of a node is the average length of the shortest paths to all other nodes [19]. Eigenvector centrality considers the centrality of a node’s neighbours [20], capturing the idea that being connected to important nodes increases one’s own importance.

Assortativity and clustering coefficient are two other structural properties of particular interest for social networks. They are typically positive [1] and have been manipulated in previous rewiring studies [5,8,10]. Correlation between the degrees of connected nodes [2] is referred to as (degree) assortativity. In social sciences, the word homophily is used to refer to the tendency of nodes that share some characteristic (degree or other attribute) to be connected to each other [18].

Clustering coefficient concerns triangles in the network. The local clustering coefficient for each node is measured as the fraction of the node’s neighbours that are also neighbours of each other [4]. Transitivity or global clustering coefficient measures the number of triangles present in the network out of all possible triangles [21].

Distances between nodes are another group of structural properties in a network. The geodesic between any pair of nodes is the shortest path to move from one to the other [22]. We measure the mean geodesic between every pair of nodes in the network, and the maximum of this distribution (referred to as the diameter).

### 2.1 Degree sequences

We selected seven empirical social networks with different relationship types from three repositories [23–25]. Their sizes ranged from 101 to 610 nodes with an average degree between 3.7 and 43 and edge density ranging from 0.006 and 0.148. The networks are:

- FilmTrust project [26] (n=101): nodes are FilmTrust project users and the relationship is trusting the film recommendations of that person.
- Scottish corporate interlock (Boards) [27] (n=131): nodes are board directors in Scotland in 1904-05 and the relationship is joint membership to a single board. We took the director projection from this bipartite network.
- French primary school [28] (n=153): nodes are primary school pupils in Marseille, France and the relationship is daily contacts (within 1 to 1.5 meters range) between them.
- Jazz collaboration [29] (n=198): nodes are Jazz band members and the relationship is playing together in a band.
- ANU Residence Hall (ANU friendship)[30] (n=217): nodes are Australia National University (ANU) students living in a residential college and the relationship was friendship.
- US Congressional Twitter [31] (n=475): nodes are members of the 117th US Congress and Senate and the relationships are interactions on Twitter (mentions, retweets, follows).
- EU institution email [31] (n=610): nodes are emails and the relationship is sending an email.

These seven empirical networks offer diverse structural property values. As expected for social networks, the average clustering coefficient is high, between 0.3 and 0.6 (see Table 1). However, the networks have low assortativity, with the highest at 0.19 and many near zero. That is inconsistent with the expected positive assortativity of social networks [1] but can be partly explained by the types of relationships where online social networks tend to have low assortativity or be disassortative [10,32]. The average shortest path length between pairs of nodes in these networks ranged between 2.1 and 5.1.

**Table 1:** Properties of empirical networks and random networks^1^ with the same degree sequence.

Network	Relationship	Density	Degree		Assortativity	Clustering Coefficient	Mean Geodesic
			Mean	Gini			
FilmTrust (N=101)	Trust recommendation	0.148	14.8	0.45	–0.12	0.53	2.144
		(0.147)	(14.7)	(0.45)	(–0.20)	(0.35)	(2.095)
Scottish Corporate Interlock (N=131)	Common membership	0.079	10.3	0.28	0.19	0.59	2.757
		(0.079)	(10.3)	(0.28)	(–0.04)	(0.12)	(2.336)
French primary school (N=153)	Proximity	0.036	5.5	0.25	0.13	0.42	4.086
		(0.036)	(5.5)	(0.25)	(0.01)	(0.05)	(3.147)
Jazz Collaboration (N=198)	Played in the same band	0.141	27.7	0.35	0.02	0.63	2.224
		(0.141)	(27.7)	(0.35)	(–0.10)	(0.25)	(1.980)
ANU Residence Hall (N=217)	Friendship	0.078	16.9	0.25	0.10	0.36	2.395
		(0.114)	(24.6)	(0.24)	(–0.04)	(0.16)	(1.974)
US Congress Twitter (N=475)	Retweet	0.091	43.0	0.30	–0.08	0.30	2.064
		(0.091)	(43.0)	(0.30)	(–0.07)	(0.16)	(1.966)
EU institution email (N=610)^2^	email exchange	0.006	3.7	0.53	0.02	0.29	5.087
		(0.006)	(3.8)	(0.52)	(–0.14)	(0.03)	(4.314)

^1^Values in brackets correspond to the average property value for the ten randomly generated networks (giant component) with the same degree sequence.

^2^The average size of the artificial networks was 587 nodes.

We used the degree sequences from each of these networks rather than the networks themselves. Within our focus of social networks, we were seeking to maximise the diversity of the degree sequences in terms of network size, density and Gini coefficient. For example, the two largest networks have very different mean degrees and the two smallest networks have very different degree variation (Table 1).

Ten random network instances were generated for each degree sequence using the Configuration model [16]. The giant component of these networks provided the starting points for the rewiring processes. The average property value over the ten artificial networks can be found in Table 1 (in brackets), with additional properties in the S1 Appendix Tables 2 and 3.

### 2.2 Rewiring processes

We used three distinct degree-preserving network rewiring algorithms that separately targeted key properties of social networks: assortativity, clustering coefficient and mean geodesic. For each algorithm, each step selected two edges and attempted to increase (assortativity, clustering coefficient) or decrease (mean geodesic) the target property. If the swap would change the target property value in the desired direction without disconnecting the network, then the swap was made.

Beyond the degree sequence, each algorithm had three additional parameters: window size, convergence, and maximum attempts. The window size is the number of attempted edge pairs swaps at which we regularly collect data during the execution of the algorithm. This is set at 20% of the number of edges in the network for the assortativity and clustering rewiring algorithms, and 10% of the edges for geodesic rewiring, regardless of the network. The convergence parameter sets the failure rate that terminates the rewiring attempts when it is no longer making progress. Convergence is set at 90% for all algorithms; that is, at least 10% of the proposed pairs during any window period must lead to a successful swap for the algorithm to continue. There is also a maximum number of attempts, set at ten times the number of edges in the network for assortativity and clustering, and at the total number of edges for geodesic rewiring algorithm.

We implemented the rewiring algorithms in Julia [33] using the *Graphs* package. All the analyses were conducted in R [34]. Source code and data can be found at OSF repository. More information can be found in the Data and Availability section.

#### 2.2.1 Assortativity rewiring.

The first algorithm focused on assortativity, the tendency of nodes with similar degrees to be connected to those with similar degrees [2]. Following other studies [5,35], we randomly selected two edges and compared the degrees of the four nodes involved. Assortativity is maximised if the two higher degree nodes are connected to each other, and similarly for the two lower degree nodes. If that differs from the existing edges, the edges are rewired to achieve this (provided this would not result in a disconnected network and that no duplicate edges would be introduced).

**Fig 1:**
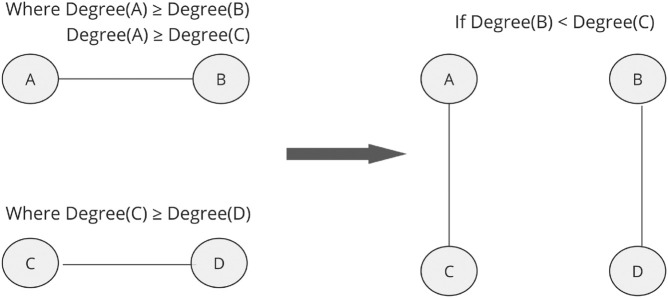
Assortativity rewiring algorithm with two pairs of nodes ((A,B) and (C,D) labelled according to degree and pairing). A rewire will be performed to pair the highest degree nodes together, if not already paired, and therefore increase the network’s assortativity.

Fig 1 shows the workings of the assortativity rewiring algorithm. The pseudocode and verification plot for this algorithm can be found in S2 Appendix Alg. 1 and the parameter values used for each degree sequence can be found in S3 Appendix Table 4.

The algorithm successfully increased assortativity for all degree sequences. The largest increase in this property was observed for the French primary school degree sequence, which increased from 0.01 to 0.85. See verification plot in S2 Appendix, Fig 17.

#### 2.2.2 Clustering coefficient rewiring.

The second algorithm targeted the average (local) clustering coefficient, the tendency of individuals to be friends with the friends of their friends [1] (and similarly for other relationships). Our algorithm adds triangles to a network by selecting a path of length five then breaking the edges at the ends of the chain and closing the triangle between the central nodes (provided that this does not produce a disconnected network or duplicated edges). This is the undirected equivalent of the algorithm used in other rewiring studies [35].

**Fig 2:**
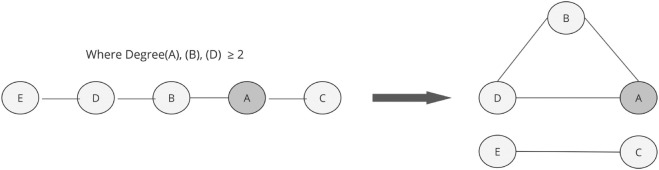
Clustering Coefficient rewiring algorithm. Node A is randomly selected and rewiring occurs if a path of length five exists and there is not already an edge connecting nodes A and D or C and E.

The path is constructed by selecting a random node with degree at least two (labelled A in Fig 2) and randomly selecting two of its incident edges. If a path of length five can be constructed (in either direction) then a potential rewire is assessed to close the triangle between the central nodes. An edge is created between the nodes at the other ends of the broken edges to preserve degree sequence. Fig 2 shows the workings of the clustering rewiring algorithm.

Like the assortativity algorithm, the rewire would only go ahead if it would not result in a disconnected network or duplicate edges. However, an additional test is needed for the clustering algorithm. The edge breaking may disrupt existing triangles, so the algorithm also checks that clustering coefficient would increase before going ahead with the rewire. The pseudocode and verification plot for this algorithm can be found in S2 Appendix, Fig 17.

This algorithm increased the clustering coefficient across all networks by between 0.1 and 0.4. As for the assortativity rewiring, the largest increase occurred for the French primary school contacts (N=153) networks, which increased the clustering coefficient from 0.05 to 0.44. See verification plot in S2 Appendix, Fig 18.

#### 2.2.3 Geodesic rewiring.

Our third algorithm targets the mean geodesic, the average length of the shortest paths between every pair of nodes in the network [22]. This algorithm decreases the average distance by directly connecting more distant pairs of nodes. The pseudocode and verification plot for this algorithm is available in the S2 Appendix Alg. 3, and S3 Appendix Table 5 provides the parameter values used for each empirical degree sequence.

**Fig 3:**
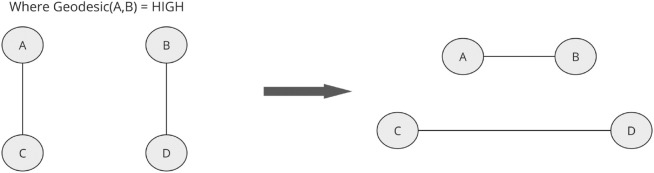
Mean geodesic rewiring algorithm example where a pair of nodes with high geodesic is chosen and a rewire is implemented if it decreases the average geodesic in the network.

The first step is to calculate the length of the shortest path between every pair of nodes. A pair of nodes is randomly selected (A and B in diagram in Fig 3) with probability proportional to their distance. For each of those nodes, select one of their neighbours with probability proportional to their degrees (C and D respectively, in Fig 3). Break the two edges, create an edge to join the original pair and another edge to join their neighbours. This preserves degrees for all four nodes. In the case where mean geodesic is increased or the rewire produces a disconnected network, the rewiring is reverted. Fig 3 show the workings of the algorithm.

Calculating shortest paths is computationally expensive and must be done twice for each attempt (before and after the proposed rewire). We chose to randomly select pairs of nodes weighted by their distance rather than simply select the most distant pairs to avoid artefacts such as zero probability of long distances in the network. However, this is also inefficient as it increases the likelihood that the rewiring would fail to decrease the average geodesic. We use a smaller window size (compared to other rewiring algorithms) of 10% of edges and set the maximum number of attempts to the number of edges so as to assess the potential of geodesic rewiring without incurring excessive computational time.

The already low mean geodesic in our starting networks (see Table 1) limited the algorithm’s effectiveness. Nonetheless, we were able to meaningfully decrease the mean geodesic of the EU institution emails networks from 4.3 to 3.8 and the French primary school networks from 3.14 to 3.04. See verification plot in S2 Appendix, Fig 19.

### 2.3 Experimental design

We generated ten random networks for each of the seven empirical degree sequences, to provide 70 networks for rewiring experiments. For each network, we ran 10 repetitions for each of the three rewiring algorithms. This produced a total of 2,100 rewiring experiments with 100 experiments for each combination of degree sequence and rewiring algorithm.

We measured a range of network properties at regular points during each rewiring experiment until rewiring was no longer successful or the experiment reached the maximum number of attempts. These regular measurements provided data concerning the simultaneous values of mean and variability of centrality measures (closeness, eigenvector, betweenness), clustering, assortativity and geodesics.

## 3 Structural property interdependencies

The analysis examined every pair of property values for each combination of rewiring mechanism and degree sequence. This section presents the property pairs where a relationship was observed for at least some degree sequences. Plots for other degree sequences can be found in the S4 Appendix (see Figs. 20 to 43). Geodesic rewiring results are included only for the EU institution emails (N=610) and the French primary school (N=153) networks as geodesics did not meaningfully decrease for the other networks.

We start by examining the relationship between the three properties targeted by our rewiring: assortativity, mean (local) clustering coefficient, and mean geodesic. This is followed by the relationship between local and global clustering coefficient, which differs by the property being targeted by rewiring. Finally, we discuss the effects of these rewiring algorithms on three centrality measures (closeness, betweenness, and eigenvector).

Degree is not considered directly as the rewiring algorithms preserve degree sequences. This means that degree is represented through different underlying empirical networks. As there are only seven degree sequences used, we have not examined the relationships between degree (mean or variation) with other properties; instead, we have noted where patterns are consistent or inconsistent across degree sequences.

### 3.1 Assortativity and clustering coefficient

Previous studies found that assortativity and mean clustering coefficient are positively related [5,6]. Our findings suggest that the relationship is more limited and depends on which property is targeted with rewiring.

**Fig 4:**
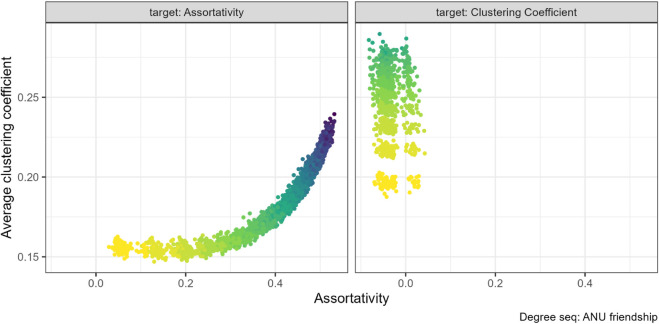
Assortativity and clustering coefficient pair combination for the ANU friendship networks (N=217). The gradient, lighter to darker, indicates the increase in number of rewiring attempts. Increasing assortativity through targeted rewiring increased the clustering coefficient.

This expected positive relationship exists when the rewiring target property is assortativity. For many of our networks increasing assortativity increased the clustering coefficient, for example for the ANU residence hall friendship networks (left panel in Fig 4).

**Fig 5:**
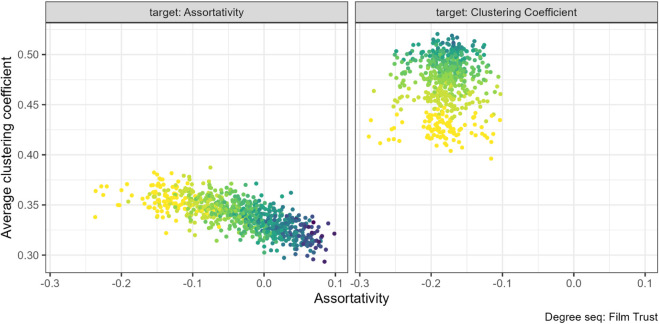
Assortativity and clustering coefficient pair combination for the FilmTrust networks (N=101). The gradient, lighter to darker, indicates the increase in number of rewiring attempts. Increasing assortativity through targeted rewiring slightly decreased the clustering coefficient.

However, we found the opposite relationship for the FilmTrust (N=101) networks, in Fig 5. As assortativity increased from negative to positive values, the clustering coefficient decreased slightly (left panel Fig 5).

On the other hand, we do not find a relationship between assortativity and clustering coefficient for any of the networks if we instead target clustering coefficient with our algorithm (right panel, Figs 4 and 5).

There was also no pattern between assortativity and clustering coefficient under the geodesic rewiring for the two degree sequences where rewiring was successful. The remaining plots for this property pair can be found at S4 Appendix (Figs. 20 to 22).

### 3.2 Assortativity and mean geodesic

**Fig 6:**
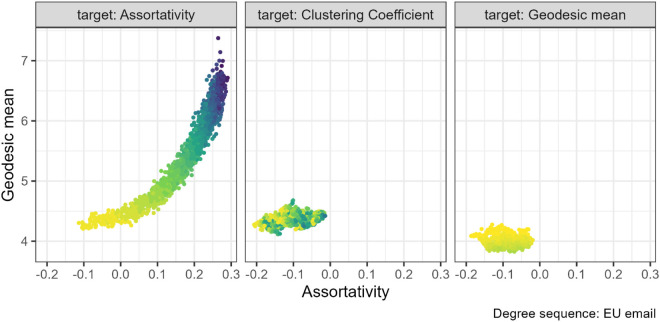
Assortativity and geodesic mean pair combination for the EU institution email networks (N=610). The gradient, lighter to darker, indicates the increase in number of rewiring attempts. Increasing assortativity through targeted rewiring increased the mean geodesic.

Previous studies have found some evidence of a positive relationship between assortativity and mean geodesic, at least for power law degree sequences and using assortativity targeted rewiring [6]. We found the same relationship across all degree sequences. With assortativity rewiring, the average path lengths in the network increase as assortativity increases. This effect is strongest for EU institution email networks (left panel Fig 6).

For most degree sequences, we were unable to sufficiently change mean geodesic with our algorithm to allow us to observe the effect on assortativity. However, even for the EU institution email networks where we successfully manipulated geodesic, there was no relationship with assortativity (right panel Fig 6).

Consistent with finding no effect on assortativity when increasing clustering coefficient, see Fig 4, there is also no pattern between assortativity and mean geodesic when rewiring for clustering coefficient. The remaining plots for this property pair can be found at S4 Appendix (Figs. 23 to 25).

### 3.3 Clustering coefficient and Mean geodesic

There is a trade-off between high clustering coefficient and low mean geodesic. When preserving degree sequence, any edge that closes a triangle is a local edge with only limited contribution to the shortest paths between all pairs of nodes. However, the Small World model [4] demonstrates the potential to substantially decrease mean geodesic in a highly clustered network with only a small number of edges that join distant nodes. We found only limited evidence of a relationship between these properties but, where the relationship is observed, it is in the expected direction of higher clustering coefficient associated with longer mean geodesics.

**Fig 7:**
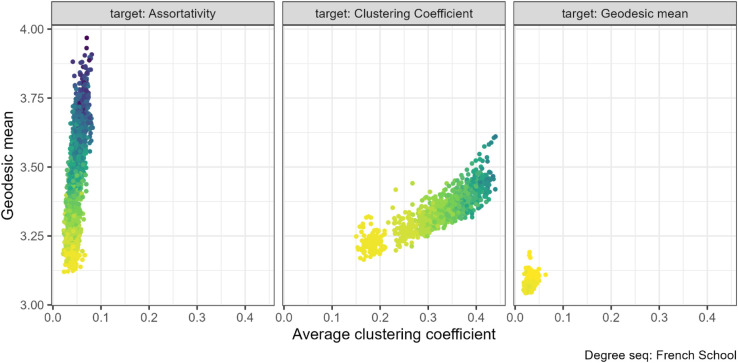
Clustering coefficient and geodesic mean pair combination for the French primary school contacts networks (N=217). The gradient, lighter to darker, indicates the increase in number of rewiring attempts. Increasing clustering coefficient through targeted rewiring had minimal or no effect on the mean geodesic.

When increasing clustering coefficient, we found no or minimal effect on mean geodesic for most networks. The strongest relationship was with the French primary school networks (N=153) (central panel Fig 7), where both properties increased. The property pair plots for the other networks can be found in the S4 Appendix (Figs. 26 to 28).

Geodesic rewiring had a very small effect on the mean geodesic for the French primary school networks (N=153), which was associated with a slight decrease in clustering coefficient (right panel Fig 7). Geodesic rewiring was more successful in reducing mean geodesic in the EU email networks but there was no clear association with the average clustering coefficient (S4 Appendix, Fig 28).

**Fig 8:**
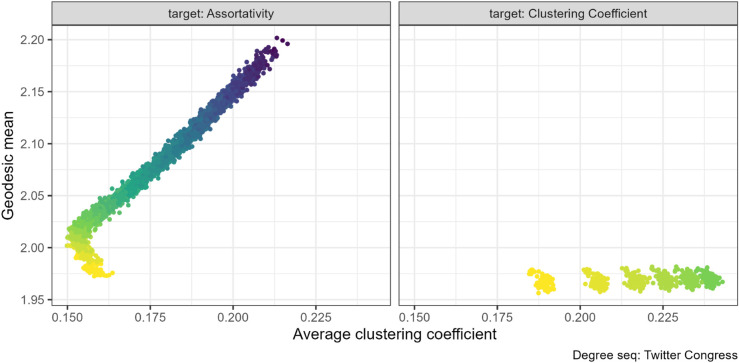
Clustering coefficient and geodesic mean pair combination for the US Congressional Twitter (N=475). Increasing assortativity through targeted rewiring increased both the clustering coefficient and the mean geodesic.

When we targeted assortativity instead, we found a positive relationship between increasing assortativity and increasing both clustering coefficient and mean geodesic. Yet, some networks showed an inverse relationship for a small section at the lower values of clustering coefficient, with a positive association later in the rewiring runs (for example, the Twitter Congress networks at Fig 8 or the Jazz networks in S4 Appendix, Fig 26. Others had no relationship, with only a small effect of assortativity on clustering coefficient or mean geodesic and consequently no pattern between these properties (such as the French primary school network, left panel Fig 7).

### 3.4 Local and global clustering coefficient

Transitivity and mean local clustering coefficient are the same property averaged at different levels. We therefore expect these properties to be positively related. While we found this to be generally true, there are some exceptions.

**Fig 9:**
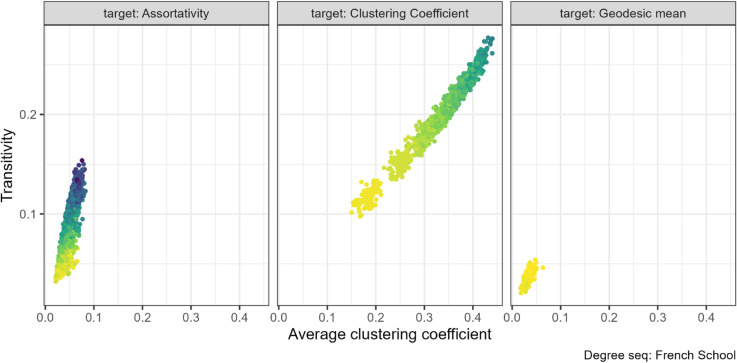
Mean (local) clustering coefficient and global clustering coefficient (transitivity) pair combination for the French primary school (N=153). The gradient, lighter to darker, indicates the increase in number of rewiring attempts. Increasing the clustering coefficient through targeted rewiring increased both the local and global clustering coefficient.

We found a positive association between local and global clustering coefficients across all seven empirical networks when the property being targeted in rewiring was the clustering coefficient, with a typical example in Fig 9 central panel, remaining plots in S4 Appendix Fig 30. Despite the identical underlying structural features, the values increase by different amounts; in this example by about 0.3 for average local and 0.2 for global.

**Fig 10:**
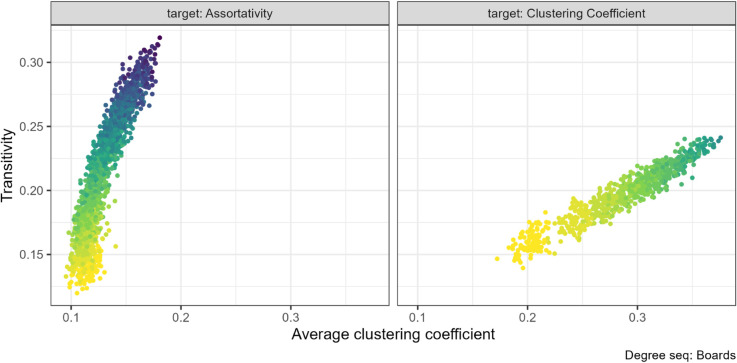
Mean (local) clustering coefficient and global clustering coefficient (transitivity) pair combination for the Scottish corporate interlock (Boards) (N=131). The gradient, lighter to darker, indicates the increase in number of rewiring attempts. Increasing the assortativity through targeted rewiring increased both the local and global clustering coefficient.

On the other hand, when we targeted assortativity, transitivity increased more than the average local clustering coefficient. The largest difference is shown in left panel of Fig 10 where globally increasing the number of triangles in the network did not increase the local clustering coefficient by the same amount. This pattern is consistent across networks, see S4 Appendix Fig 29.

**Fig 11:**
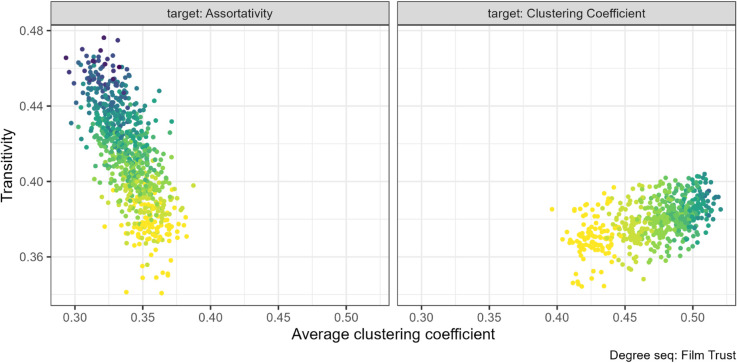
Mean (local) clustering coefficient and global clustering coefficient (transitivity) pair combination for the FilmTrust project (N=101). The gradient, lighter to darker, indicates the increase in number of rewiring attempts. Increasing the assortativity through targeted rewiring decreased the clustering coefficient and slightly increased the transitivity.

Furthermore, for the FilmTrust project networks, we previously noted that the assortativity rewiring decreased the average (local) clustering coefficient (see Fig 4). Yet, we find that as assortativity increased, the network transitivity increased slightly (see left panel Fig 11). Thus, showing an inverse relationship between these two properties for this degree sequence.

Lastly, for the two degree sequences where geodesic rewiring was successful, we found slight evidence of a positive association between transitivity and mean local clustering coefficient. However, the change in both properties was very small. The plots showing local and global clustering coefficient for the remaining five social networks can be found in the S4 Appendix Fig 31.

### 3.5 Centrality measures

We observed relationships between the properties targeted by our rewiring algorithms and both the mean and variation in various centrality measures. Our algorithms are degree preserving, so the mean degree and Gini coefficient of degree do not change. Instead, we measured the distributions of three other node centralities: closeness, betweenness, and eigenvector.

**Fig 12:**
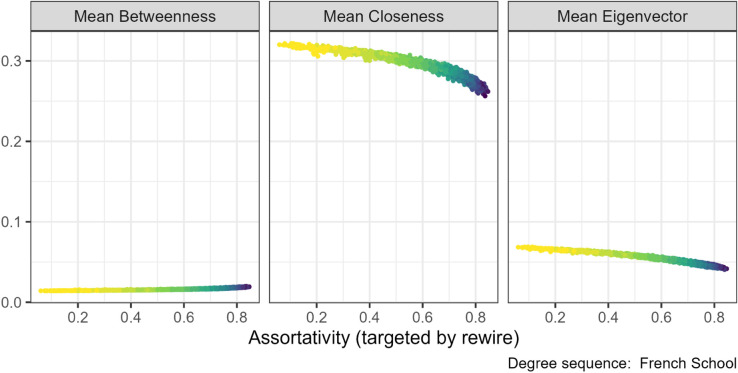
Centrality measures under the assortativity targeted rewiring for the French primary school (N=153). The gradient, lighter to darker, indicates the increase in number of rewiring attempts. Increasing the assortativity through targeted rewiring slightly decreased the mean closeness centrality but not the mean betweenness or eigenvector centralities.

When we increased assortativity, there was a slight decrease in the mean closeness (central panel Fig 12) and an associated increase in the Gini of closeness. Mean eigenvector centrality (right panel Fig 12) also decreased but to a lesser extent. Mean betweenness is unaffected and remains near zero (left panel Fig 12). However, Gini betweenness decreased substantially, which is opposite to the direction of the other centrality measures. This pattern was consistent across all seven degree sequences, see S4 Appendix Figs. 32, 38, 44 for other plots.

**Fig 13:**
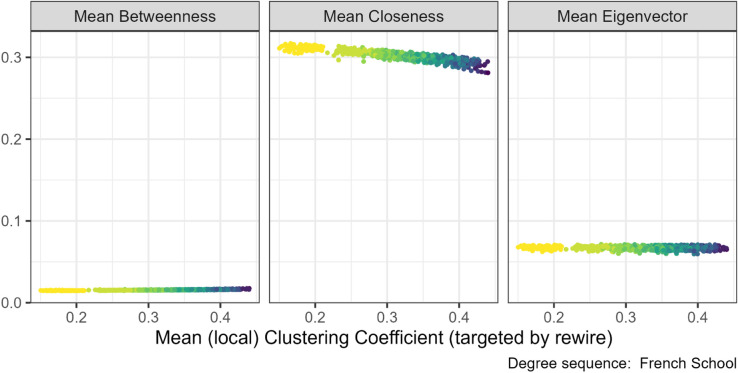
Centrality measures under the clustering coefficient targeted rewiring for French primary school networks (N=153). The gradient, lighter to darker, indicates the increase in number of rewiring attempts. Increasing the clustering coefficient through targeted rewiring slightly decreased the mean closeness centrality but not the mean betweenness or eigenvector centralities.

Increasing clustering coefficient was associated with a small decrease in mean closeness centrality under two of the degree sequences, French School (see central panel Fig 13) and Boards networks (in S4 Appendix, Fig 33), along with a very small increase in Gini closeness. For the other experiments, any relationship was very weak but appeared to be in the same direction. The rest of the plots can be found in the S4 Appendix Fig 33. No effect was found on the other two centrality measures, see S4 Appendix Figs. 39 and 45.

**Fig 14:**
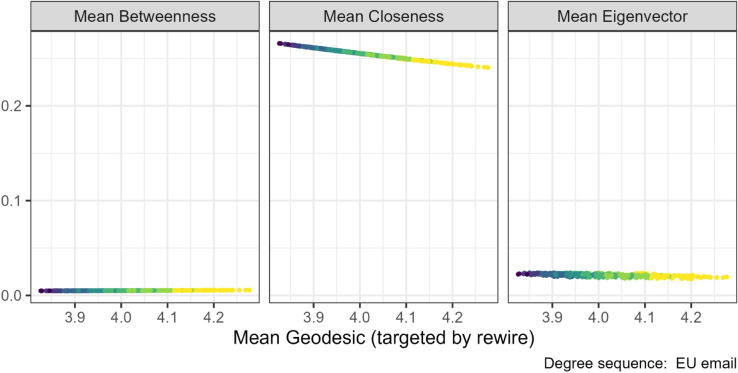
Centrality measures under the geodesic mean targeted rewiring for the Emails inside EU institution (N=610). The gradient, lighter to darker, indicates the increase in number of rewiring attempts. Decreasing the mean geodesic through targeted rewiring slightly increased the mean closeness centrality but not the mean betweenness or eigenvector centralities.

Our geodesic rewiring decreases distances in the network and can therefore be expected to also increase closeness. This association was observed in the EU institution email degree sequence (central panel Fig 14), which are the networks where rewiring had the strongest effect. Also, the Gini betweenness increased. We found no relationship between geodesic and the other centrality measures (left and right panels Fig 14).

**Fig 15:**
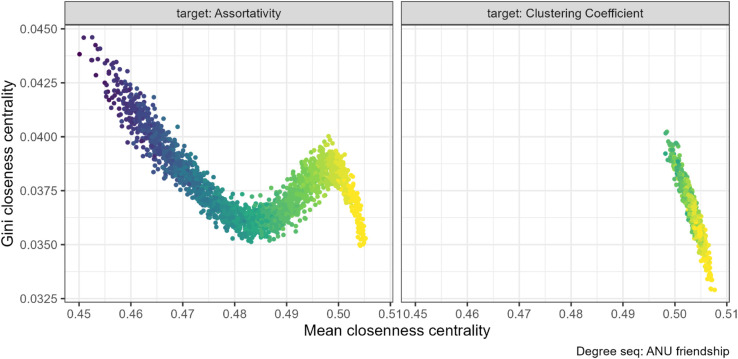
Mean and Gini closeness centrality pair combination for the ANU residence hall (N=217) network. The gradient, lighter to darker, indicates the increase in number of rewiring attempts. Increasing the assortativity through targeted rewiring (left panel) slightly decreased the mean betweenness centrality and increased the Gini betweenness centrality. Increasing the clustering coefficient (right panel) decreased the mean betweenness centrality and increased the Gini betweenness centrality.

Decreased mean and increased inequality in the node centrality measures combine for a generally negative association between these properties. For many of the relationships, changes are consistent but very small. For three of the sequences, the relationship is reversed for part of the property space, as can be seen in Fig 15 for the ANU residence networks. See S4 Appendix for remaining networks under different rewiring algorithms 35 to 37.

**Fig 16:**
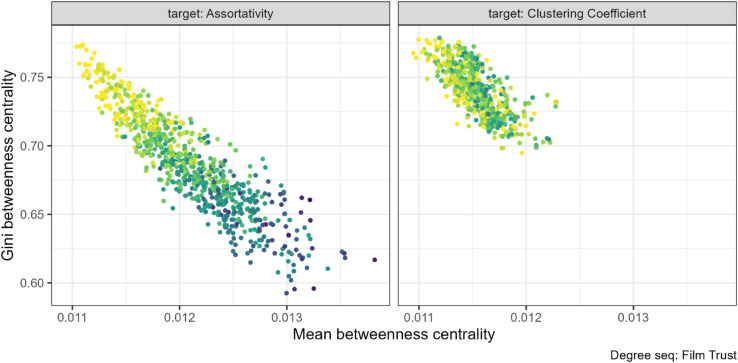
Mean betweenness and Gini betweenness pair combination for the FilmTrust (N=101) network. The gradient, lighter to darker, indicates the increase in number of rewiring attempts. Increasing the assortativity through target rewiring (left panel) slightly increased the mean betweenness centrality and decreased the Gini betweenness centrality. Note that the change in mean is extremely small. No relationship was found under the clustering coefficient target rewiring (right panel) between these properties.

The association between mean and Gini of betweenness is also negative (see Fig 16 for the FilmTrust sequences). While the relationship is identical, the two properties have responded in opposite ways as assortativity increases. See S4 Appendix for remaining networks Figs. 41 to 43.

## 4 Discussion

Social networks are characterised by skewed degree distributions, relatively short geodesics, positive degree assortativity and mean clustering coefficients. Previous literature has identified relationships between these properties, typically in the context of artificial or other networks that are not social networks. Here instead we used degree sequences from social networks and expand the properties measured to understand these relationships more systematically, as small-sized social networks have received limited attention in the literature. Within the space of these social networks, we selected degree sequences that are diverse in terms of network size, density and Gini coefficient.

We found positive associations between assortativity, clustering coefficient and geodesics for our networks, consistent with [8] findings on Facebook friendship networks and [5,6] findings in synthetic and biological networks. However, the relationships we found between geodesics and the other properties were weaker, partly explained by the small size of our networks and consequent short path lengths.

Our results suggest that the relationship between assortativity and clustering coefficient is more complex than previously understood, potentially influenced by the mechanics of the rewiring and the specific values of assortativity and clustering coefficient achievable by the degree sequence, as well as degree heterogeneity. We found that increasing assortativity also increases clustering coefficient, but that the opposite is not true. The relationship is stronger at assortativity values above about 0.3. This conflicts with [10], who found that increasing clustering coefficient also induces an increase in assortativity, but their simulated networks had zero assortativity and clustering coefficient unlike our social networks. In our case, where the degree distribution is broad (have higher degree heterogeneity), like the FilmTrust and EU email degree sequences, it showed minimal (EU email) or negative (FilmTrust) impact on clustering coefficient as assortativity increase.

Previous work and our results have found a positive relationship between both measures of triad closure in networks, local and global clustering coefficient. Moreover, local clustering coefficient, is typically higher than transitivity [36] and it increases faster under manipulation [8]. We also found that local clustering coefficient increased faster than transitivity when targeting clustering, but the opposite generally occurred when targeting assortativity.

We also found that the three network centrality measures (closeness, node betweenness and eigenvector) were somewhat unresponsive to manipulations of assortativity, clustering coefficient and mean geodesic. This is consistent with the degree preserving results from [37] applied to various types of networks, including social networks (null1 models in their paper). There was a small positive relationship between assortativity and mean closeness and negative relationship with inequality of closeness. The weak relationships we found between targeted properties and betweenness were in the opposite direction, consistent with [12] that betweenness responds differently than other centrality measures.

There are some limitations to this research. We took diverse degree sequences of small social networks in terms of network density and Gini coefficient and found inconsistent associations even within that set of degree sequences which limits the generalisability of our results. Relationships may not hold with larger networks or other degree sequences.

Overall, our findings on small-size social networks highlight the complexity of the relationships between the important network properties of degree heterogeneity, assortativity, clustering coefficient, and geodesics. Relationships may be conditional on other network properties or restricted to particular areas of the property space.

## Supporting information

SI Appendix 1 Additional structural prosperities.
(PDF)

SI Appendix 2 Pseudocode.
(PDF)

SI Appendix 3 Experimental conditions.
(PDF)

SI Appendix 4 Additional property interaction pairs.
(PDF)
