# Correction: The importance of structure: Using targeted rewiring to explore social networks property interdependencies

**DOI:** 10.1371/journal.pone.0347498

**Published:** 2026-04-15

**Authors:** Cristina Chueca Del Cerro, Jennifer Badham

SI Appendix 1–4 were published without the plots and supplementary labels for the tables. It can be viewed below.

## Supporting information

S1 Appendix Additional structural properties. (PDF)

S2 Appendix Pseudocode. (PDF)

S3 Appendix Experimental conditions. (PDF)

S4 Appendix Additional property interaction pairs. (PDF)
