# Supplementary material for: The importance of structure: Using targeted rewiring to explore social networks property interdependencies
Source: PLoS One. 2026 Mar 20;21(3):e0336496. doi: 10.1371/journal.pone.0336496 (PMC13004504; doi:10.1371/journal.pone.0336496)
Supplement: SI Appendix 1 — Additional structural prosperities. (PDF) [file pone.0336496.s001.pdf]

The importance of structure: using targeted rewiring to explore social networks property interdependencies

Cristina Chueca Del Cerro[1*] Jennifer Badham[1]

1 Department of Sociology, Durham University, Durham, United Kingdom

* Cristina.Chueca-Del-Cerro@durham.ac.uk

Abstract

Social networks typically have skewed degree distributions and relatively high clustering and assortativity coefficients. Some studies have explored the relationships between these properties, but have given limited attention to social networks and have found conflicting evidence. To expand our understanding of the ways that properties constrain each other in social networks we use separate degree-preserving rewiring algorithms to manipulate assortativity, clustering coefficient and mean geodesic of networks constructed from seven diverse empirical degree sequences. We measured centrality (mean and Gini coefficient of several measures), clustering, assortativity and network distances. Only a small number of property pairs showed a relationship. Further, where interdependencies do exist, they are conditional and occur only for specific value ranges or a subset of the tested networks.

A Additional structural properties

This appendix includes the rest of the structural properties we measured for the empirical networks, not shown in Table **??**. These can be found in Tables 1 and 2.

Table 1. Centrality measures for empirical networks and corresponding averaged artificial networks, in brackets

Network	Closeness		Betweenness		Eigenvector	
	Mean	Gini	Mean	Gini	Mean	Gini
FilmTrust	0.47	0.09	0.012	0.73	0.29	0.45
(N=101)	(0.48)	(0.08)	(0.011)	(0.75)	(0.08)	(0.44)
Scottish Corporate interlock	0.37	0.08	0.014	0.65	0.21	0.48
(N=131)	(0.43)	(0.05)	(0.010)	(0.50)	(0.07)	(0.35)
French School	0.25	0.07	0.020	0.54	0.24	0.51
(N=153)	(0.32)	(0.06)	(0.014)	(0.44)	(0.07)	(0.35)
Jazz Collaboration	0.46	0.006	0.01	0.72	0.27	0.44
(N=198)	(0.51)	(0.05)	(0.005)	(0.62)	(0.06)	(0.36)
ANU friendships	0.42	0.05	0.006	0.53	0.26	0.36
(N=217)	(0.51)	(0.03)	(0.005)	(0.47)	(0.06)	(0.27)
US Congress Twitter	0.49	0.04	0.002	0.59	0.21	0.31
(N=475)	(0.51)	(0.03)	(0.002)	(0.58)	(0.04)	(0.33)
EU institution email	0.20	0.11	0.007	0.82	0.05	0.79
(N=610)	(0.24)	(0.09)	(0.006)	(0.79)	(0.02)	(0.69)

Table 2. Additional structural properties for empirical networks and corresponding averaged artificial networks, in brackets

Network	Clustering Coeff		Transitivity	Geodesic		Diameter
	Mean	Gini		Mean	Gini	
FilmTrust	0.53	0.279	0.42	2.165	0.17	5
(N=101)	(0.35)	(0.33)	(0.36)	(2.095)	(0.16)	(4)
Scottish Corporate interlock	0.59	0.200	0.50	2.757	0.20	6
(N=131)	(0.12)	(0.42)	(0.13)	(2.336)	(0.15)	(4)
French School	0.424	0.352	0.36	4.090	0.20	9
(N=153)	(0.05)	(0.77)	(0.05)	(3.147)	(0.16)	(6)
Jazz Collaboration	0.63	0.181	0.52	2.235	0.18	6
(N=198)	(0.25)	(0.23)	(0.26)	(1.980)	(0.12)	(4)
ANU friendship	0.36	0.195	0.52	2.395	0.14	4
(N=217)	(0.16)	(0.19)	(0.16)	(1.974)	(0.10)	(4)
US Congress Twitter	0.30	0.167	0.27	2.064	0.11	4
(N=475)	(0.16)	(0.20)	(0.16)	(1.966)	(0.07)	(4)
EU institution email	0.29	0.779	0.19	5.089	0.19	13
(N=610)	(0.03)	(0.93)	(0.05)	(4.31)	(0.16)	(10)
