# Supplementary material for: The importance of structure: Using targeted rewiring to explore social networks property interdependencies
Source: PLoS One. 2026 Mar 20;21(3):e0336496. doi: 10.1371/journal.pone.0336496 (PMC13004504; doi:10.1371/journal.pone.0336496)
Supplement: SI Appendix 2 — Pseudocode. (PDF) [file pone.0336496.s002.pdf]

The importance of structure: using targeted rewiring to explore social networks property interdependencies

Cristina Chueca Del Cerro[1*] Jennifer Badham[1]

1 Department of Sociology, Durham University, Durham, United Kingdom

* Cristina.Chueca-Del-Cerro@durham.ac.uk

Abstract

Social networks typically have skewed degree distributions and relatively high clustering and assortativity coefficients. Some studies have explored the relationships between these properties, but have given limited attention to social networks and have found conflicting evidence. To expand our understanding of the ways that properties constrain each other in social networks we use separate degree-preserving rewiring algorithms to manipulate assortativity, clustering coefficient and mean geodesic of networks constructed from seven diverse empirical degree sequences. We measured centrality (mean and Gini coefficient of several measures), clustering, assortativity and network distances. Only a small number of property pairs showed a relationship. Further, where interdependencies do exist, they are conditional and occur only for specific value ranges or a subset of the tested networks.

1 Pseudocode

This appendix includes the pseudocode for each of the three rewiring algorithms (see 1 to 3) and corresponding verification plot demonstrating their effectiveness in manipulating each property individually (see Figures 1, 2 and 3).

Algorithm 1 Pseudocode Assortativity Algorithm

```
Input: Network Edgelist

while Convergence & Max attempts ≤ threshold do
    Sample two unique pairs of edges from graph
    Label highest degree as A, label B its partner
    Label highest degree of second edge as C, label D its partner
    Order them by their degree (A,B,C,D) OR (A,C,B,D) by definition
    if (A,C,B,D) then
        Break original edges (A,B) (C,D)
        Create edges (A,C) (B,D)
        if Disconnected OR Assortativity does not increase then
            Reverse edge creation
            Failed rewire
        else
            Successful rewire
        end if
    else
        Failed rewire
    end if
    if mod(attempts, window) = 0 then
        Calculate network properties
    end if
end while

Output: Rewired Network
```

Fig 1. Verification plot for the assortativity rewiring algorithm showing changes in this property across all seven degree sequences. The grey area represents the 25th and 75th percentiles. As the number of rewiring attempts increases, the network's assortativity increases.

Fig 2. Verification plot for the average clustering coefficient rewiring algorithm showing changes in this property across all seven degree sequences. The grey area represents the 25th and 75th percentiles. As the number of rewiring attempts increases, the network's average clustering coefficient increases.

Fig 3. Verification plot for the mean geodesic rewiring algorithm showing changes in this property across all seven degree sequences. The grey area represents the 25th and 75th percentiles. As the number of rewiring attempts increases, the network's mean geodesic decreases.

Algorithm 2 Pseudocode (Local) Clustering Coefficient Rewiring Algorithm

```
Input: Network Edgelist

while Convergence & Max attempts ≤ threshold do
  Select random node A with degree ≥ 2
  Sample two neighbours (B,C) such that B has degree ≥ 2 (A,B) (A,C)
  if No suitable B neighbour then
    Failed rewire
  else
    Sample D, a neighbour of B, with degree ≥ 2 AND D =/ A
    Sample E, a neighbour of D AND E =/ B
  end if
  Break edges (A,C) AND (D,E)
  Create edges (A,D) AND (C,E)
  if Disconnected OR does not increase clustering coefficient then
    Reverse edge creation
    Failed rewire
  else
    Successful rewire
  end if
  if mod(attempts, window) = 0 then
    Calculate network properties
  end if
end while

Output: Rewired Network
```

Algorithm 3 Pseudocode Geodesic Mean Rewiring Algorithm

Input: Network Edgelist

```
while Convergence & Max attempts ≤ threshold do
    Calculate the geodesic for all the nodes
    Sample two nodes with HIGH geodesic (A,B)
    Sample one neighbour for each node (C and D respectively), probability proportional to degree
    Break edges (A,C) (B,D)
    Create edges (A,B) (C,D)
    if Disconnected OR Geodesic mean does not decrease then
        Reverse edge creation
        Failed rewire
    else
        Successful rewire
    end if
    if mod(attempts, window) = 0 then
        Calculate network properties
    end if
end while
```

Output: Rewired Network
