# Supplementary material for: The importance of structure: Using targeted rewiring to explore social networks property interdependencies
Source: PLoS One. 2026 Mar 20;21(3):e0336496. doi: 10.1371/journal.pone.0336496 (PMC13004504; doi:10.1371/journal.pone.0336496)
Supplement: SI Appendix 3 — Experimental conditions. (PDF) [file pone.0336496.s003.pdf]

The importance of structure: using targeted rewiring to explore social networks property interdependencies

Cristina Chueca Del Cerro[1*] Jennifer Badham[1]

1 Department of Sociology, Durham University, Durham, United Kingdom

* Cristina.Chueca-Del-Cerro@durham.ac.uk

Abstract

Social networks typically have skewed degree distributions and relatively high clustering and assortativity coefficients. Some studies have explored the relationships between these properties, but have given limited attention to social networks and have found conflicting evidence. To expand our understanding of the ways that properties constrain each other in social networks we use separate degree-preserving rewiring algorithms to manipulate assortativity, clustering coefficient and mean geodesic of networks constructed from seven diverse empirical degree sequences. We measured centrality (mean and Gini coefficient of several measures), clustering, assortativity and network distances. Only a small number of property pairs showed a relationship. Further, where interdependencies do exist, they are conditional and occur only for specific value ranges or a subset of the tested networks.

1 Experimental conditions

This appendix includes the experimental conditions (window size and maximum attempts) for each of the three rewiring algorithms, see Tables 1- 2.

Table 1. Experimental conditions for the assortativity and clustering coefficient rewiring algorithms

Network	Window	Max attempts
FilmTrust (N=101)	149	7,450
Scottish Corporate interlock (N=131)	135	6,760
French School (N=153)	84	4,210
Jazz Collaboration (N=198)	548	27,420
ANU friendship (N=217)	534	26,710
US Congress Twitter (N=475)	2,044	102,220
EU institution email (N=610)	224	11,190

Table 2. Experimental conditions for the geodesic mean rewiring algorithm.

Network	Window	Max attempts
FilmTrust (N=101)	75	7,450
Scottish Corporate interlock (N=131)	67	6,760
French School (N=153)	42	4,210
Jazz Collaboration (N=198)	274	27,420
ANU friendship (N=217)	267	26,710
US Congress Twitter (N=475)	1,022	102,220
EU institution email (N=610)	112	11,190
