# Supplementary material for: Correction: The importance of structure: Using targeted rewiring to explore social networks property interdependencies
Source: PLoS One. 2026 Apr 15;21(4):e0347498. doi: 10.1371/journal.pone.0347498 (PMC13082640; doi:10.1371/journal.pone.0347498)
Supplement: S1 Appendix — (PDF) [file pone.0347498.s001.pdf]

Additional structural properties

This appendix includes the rest of the structural properties we measured for the empirical networks, not shown in Table 1. These can be found in Tables S1.1 and S1.2.

Table S1.1: Centrality measures for empirical networks and corresponding averaged artificial networks, in brackets

**Network**	**Closeness**		**Betweenness**		**Eigenvector**	
	**Mean**	**Gini**	**Mean**	**Gini**	**Mean**	**Gini**
FilmTrust	0.47	0.09	0.012	0.73	0.29	0.45
(N=101)	(0.48)	(0.08)	(0.011)	(0.75)	(0.08)	(0.44)
Scottish Corporate interlock	0.37	0.08	0.014	0.65	0.21	0.48
(N=131)	(0.43)	(0.05)	(0.010)	(0.50)	(0.07)	(0.35)
French School	0.25	0.07	0.020	0.54	0.24	0.51
(N=153)	(0.32)	(0.06)	(0.014)	(0.44)	(0.07)	(0.35)
Jazz Collaboration	0.46	0.006	0.01	0.72	0.27	0.44
(N=198)	(0.51)	(0.05)	(0.005)	(0.62)	(0.06)	(0.36)
ANU friendships	0.42	0.05	0.006	0.53	0.26	0.36
(N=217)	(0.51)	(0.03)	(0.005)	(0.47)	(0.06)	(0.27)
US Congress Twitter	0.49	0.04	0.002	0.59	0.21	0.31
(N=475)	(0.51)	(0.03)	(0.002)	(0.58)	(0.04)	(0.33)
EU institution email	0.20	0.11	0.007	0.82	0.05	0.79
(N=610)	(0.24)	(0.09)	(0.006)	(0.79)	(0.02)	(0.69)

Table S1.2: Additional structural properties for empirical networks and corresponding averaged artificial networks, in brackets

**Network**	**Clustering Coeff**		**Transitivity**	**Geodesic**		**Diameter**
	**Mean**	**Gini**		**Mean**	**Gini**	
FilmTrust	0.53	0.279	0.42	2.165	0.17	5
(N=101)	(0.35)	(0.33)	(0.36)	(2.095)	(0.16)	(4)
Scottish Corporate interlock	0.59	0.200	0.50	2.757	0.20	6
(N=131)	(0.12)	(0.42)	(0.13)	(2.336)	(0.15)	(4)
French School	0.424	0.352	0.36	4.090	0.20	9
(N=153)	(0.05)	(0.77)	(0.05)	(3.147)	(0.16)	(6)
Jazz Collaboration	0.63	0.181	0.52	2.235	0.18	6
(N=198)	(0.25)	(0.23)	(0.26)	(1.980)	(0.12)	(4)
ANU friendship	0.36	0.195	0.52	2.395	0.14	4
(N=217)	(0.16)	(0.19)	(0.16)	(1.974)	(0.10)	(4)
US Congress Twitter	0.30	0.167	0.27	2.064	0.11	4
(N=475)	(0.16)	(0.20)	(0.16)	(1.966)	(0.07)	(4)
EU institution email	0.29	0.779	0.19	5.089	0.19	13
(N=610)	(0.03)	(0.93)	(0.05)	(4.31)	(0.16)	(10)
