# Supplementary material for: Correction: The importance of structure: Using targeted rewiring to explore social networks property interdependencies
Source: PLoS One. 2026 Apr 15;21(4):e0347498. doi: 10.1371/journal.pone.0347498 (PMC13082640; doi:10.1371/journal.pone.0347498)
Supplement: S2 Appendix — (PDF) [file pone.0347498.s002.pdf]

Pseudocode

This appendix includes the pseudocode for each of the three rewiring algorithms and corresponding verification plot demonstrating their effectiveness in manipulating each property individually (see Figures 17 to 19).

Algorithm 1 Pseudocode Assortativity Algorithm

```
Input: Network Edgelist

while Convergence & Max attempts ≤ threshold do
    Sample two unique pairs of edges from graph
    Label highest degree as A, label B its partner
    Label highest degree of second edge as C, label D its partner
    Order them by their degree (A,B,C,D) OR (A,C,B,D) by definition
    if (A,C,B,D) then
        Break original edges (A,B) (C,D)
        Create edges (A,C) (B,D)
        if Disconnected OR Assortativity does not increase then
            Reverse edge creation
            Failed rewire
        else
            Successful rewire
        end if
    else
        Failed rewire
    end if
    if mod(attempts, window) = 0 then
        Calculate network properties
    end if
end while

Output: Rewired Network
```

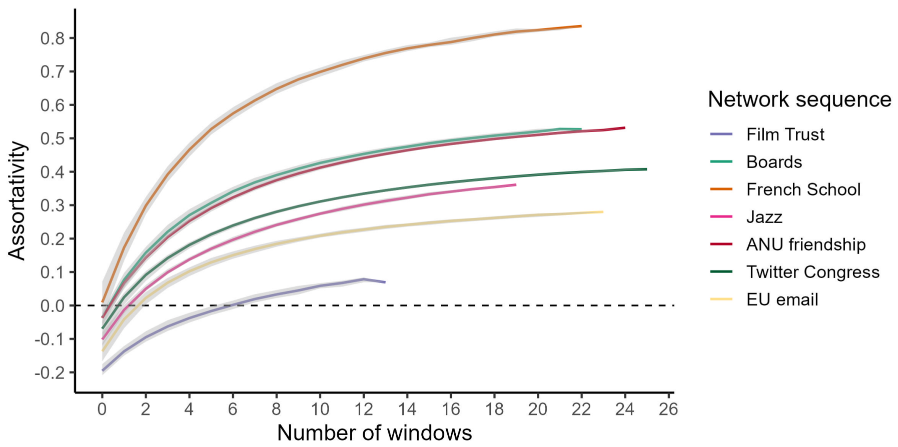

Figure 17: Verification plot for the assortativity rewiring algorithm showing changes in this property across all seven degree sequences. The grey area represents the 25th and 75th percentiles. As the number of rewiring attempts increases, the network's assortativity increases.

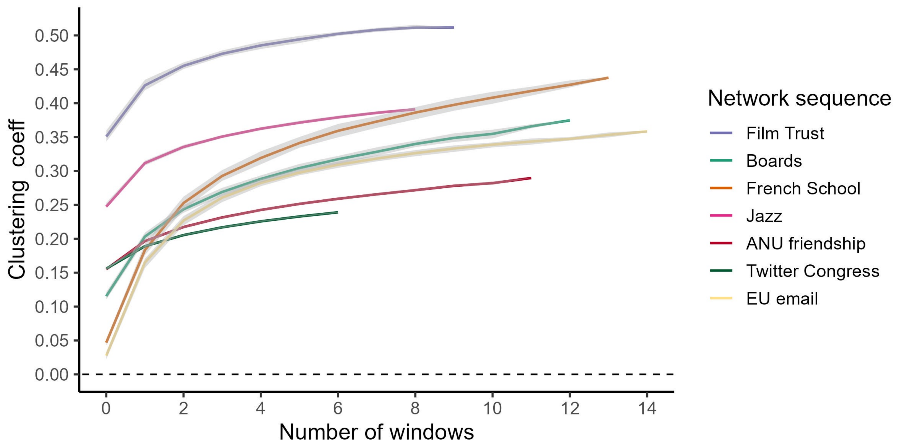

Figure 18: Verification plot for the average clustering coefficient rewiring algorithm showing changes in this property across all seven degree sequences. The grey area represents the 25th and 75th percentiles. As the number of rewiring attempts increases, the network's average clustering coefficient increases.

Algorithm 2 Pseudocode (Local) Clustering Coefficient Rewiring Algorithm

```
Input: Network Edgelist

while Convergence & Max attempts ≤ threshold do
  Select random node A with degree ≥ 2
  Sample two neighbours (B,C) such that B has degree ≥ 2 (A,B) (A,C)
  if No suitable B neighbour then
    Failed rewire
  else
    Sample D, a neighbour of B, with degree ≥ 2 AND D =/ A
    Sample E, a neighbour of D AND E =/ B
  end if
  Break edges (A,C) AND (D,E)
  Create edges (A,D) AND (C,E)
  if Disconnected OR does not increase clustering coefficient then
    Reverse edge creation
    Failed rewire
  else
    Successful rewire
  end if
  if mod(attempts, window) = 0 then
    Calculate network properties
  end if
end while

Output: Rewired Network
```

Algorithm 3 Pseudocode Geodesic Mean Rewiring Algorithm

Input: Network Edgelist

while Convergence & Max attempts $\leq$ threshold **do**
  Calculate the geodesic for all the nodes
  Sample two nodes with HIGH geodesic (A,B)
  Sample one neighbour for each node (C and D respectively), probability proportional to degree
  Break edges (A,C) (B,D)
  Create edges (A,B) (C,D)
  if Disconnected OR Geodesic mean does not decrease **then**
    Reverse edge creation
    Failed rewire
  else
    Successful rewire
  end if
  if mod(attempts, window) = 0 **then**
    Calculate network properties
  end if
end while

Output: Rewired Network

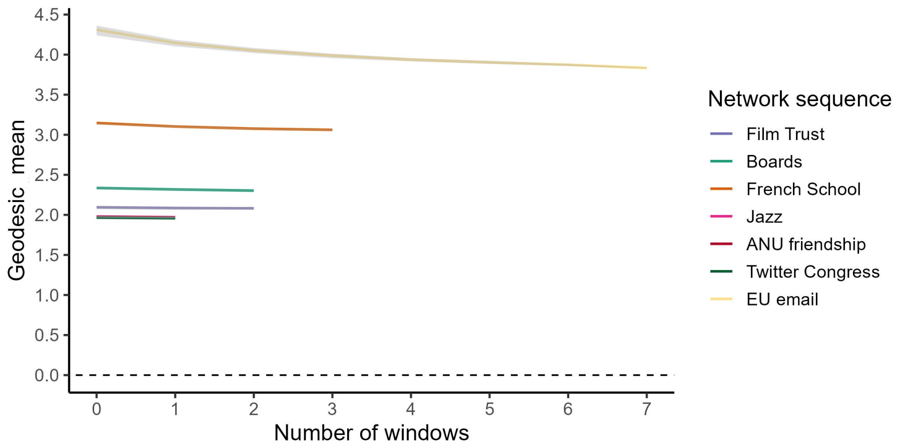

Figure 19: Verification plot for the mean geodesic rewiring algorithm showing changes in this property across all seven degree sequences. The grey area represents the 25th and 75th percentiles. As the number of rewiring attempts increases, the network's mean geodesic decreases.
