# Supplementary material for: Correction: The importance of structure: Using targeted rewiring to explore social networks property interdependencies
Source: PLoS One. 2026 Apr 15;21(4):e0347498. doi: 10.1371/journal.pone.0347498 (PMC13082640; doi:10.1371/journal.pone.0347498)
Supplement: S3 Appendix — (PDF) [file pone.0347498.s003.pdf]

Experimental conditions

This appendix includes the experimental conditions (window size and maximum attempts) for each of the three rewiring algorithms, see Tables S3.1 and S3.2.

Table S3.1: Experimental conditions for the assortativity and clustering coefficient rewiring algorithms

**Network**	**Window**	**Max attempts**
FilmTrust (N=101)	149	7,450
Scottish Corporate interlock (N=131)	135	6,760
French School (N=153)	84	4,210
Jazz Collaboration (N=198)	548	27,420
ANU friendship (N=217)	534	26,710
US Congress Twitter (N=475)	2,044	102,220
EU institution email (N=610)	224	11,190

Table S3.2: Experimental conditions for the geodesic mean rewiring algorithm.

**Network**	**Window**	**Max attempts**
FilmTrust (N=101)	75	7,450
Scottish Corporate interlock (N=131)	67	6,760
French School (N=153)	42	4,210
Jazz Collaboration (N=198)	274	27,420
ANU friendship (N=217)	267	26,710
US Congress Twitter (N=475)	1,022	102,220
EU institution email (N=610)	112	11,190
