# Supplementary material for: Correction: The importance of structure: Using targeted rewiring to explore social networks property interdependencies
Source: PLoS One. 2026 Apr 15;21(4):e0347498. doi: 10.1371/journal.pone.0347498 (PMC13082640; doi:10.1371/journal.pone.0347498)
Supplement: S4 Appendix — (PDF) [file pone.0347498.s004.pdf]

Additional property interaction pairs plots

This appendix reports all property interaction pairs plots for each of the degree sequences and rewiring algorithms. We have three figures per property pair combination, each plot corresponds to a specific rewiring algorithm for all networks.

Assortativity and Clustering coefficient

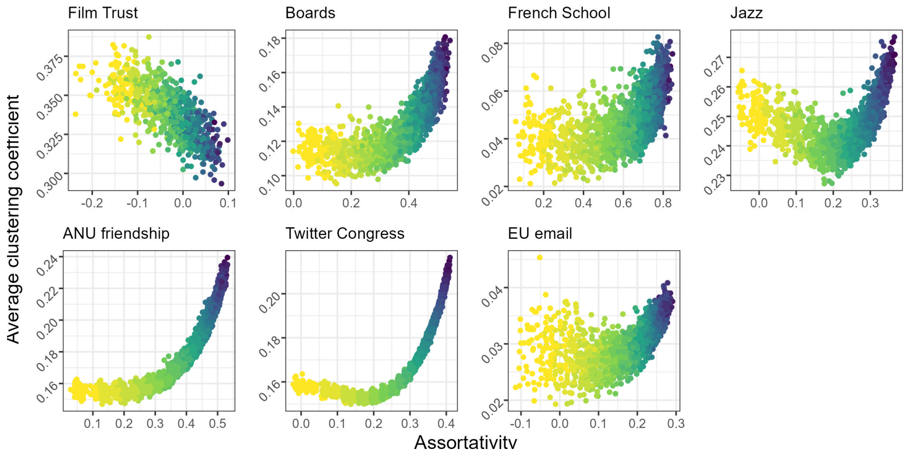

Figure 20: Assortativity and clustering relationship with assortativity rewiring. The ANU Residence networks also appear at Fig. 4 as showing the strongest relationship. FilmTrust, also shown at Fig. 5, is atypical. The gradient, lighter to darker, indicates the increase in number of rewiring attempts. Increasing assortativity increased the clustering coefficient, except for the FilmTrust where it slightly decreased the clustering coefficient.

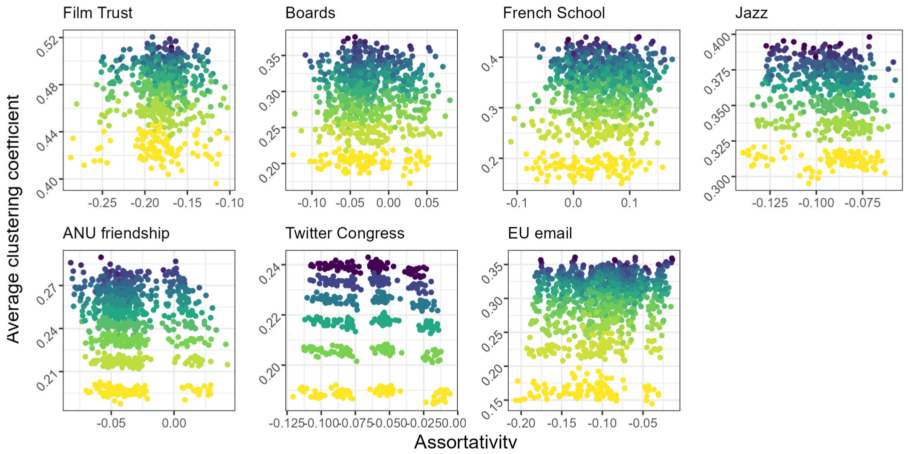

Figure 21: Assortativity and Clustering coefficient pair combination with clustering coefficient rewiring. The ANU Residence and FilmTrust were shown at Figs 4 - 5, demonstrating the lack of relationship. The gradient, lighter to darker, indicates the increase in number of rewiring attempts. We found no relationship between increasing the clustering coefficient through targeted rewiring and changes in the assortativity for any of the networks.

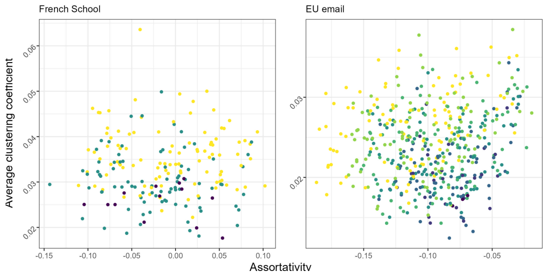

Figure 22: Assortativity and Clustering coefficient pair combination with mean geodesic rewiring. No pattern was observed for either of the two degree sequences. The gradient, lighter to darker, indicates the increase in number of rewiring attempts. We found no relationship between decreasing the mean geodesic and changes in the assortativity for any of the networks.

Assortativity and geodesic mean

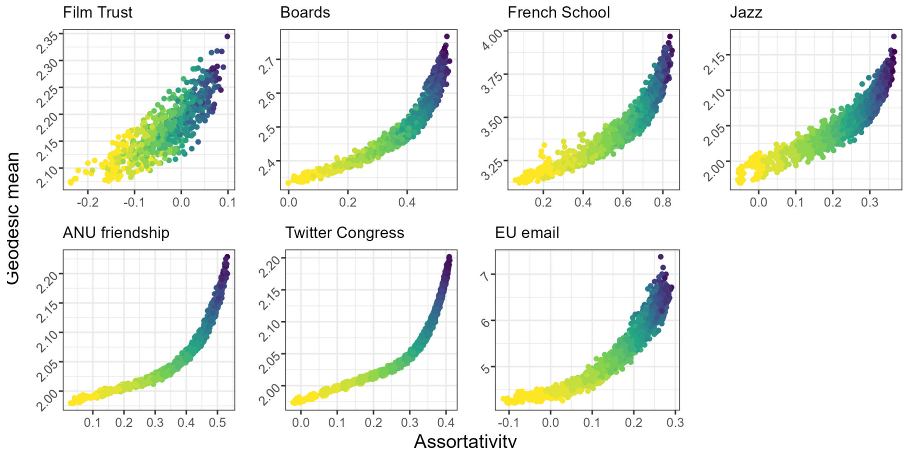

Figure 23: Assortativity and Geodesic mean pair combination with assortativity rewiring. The EU Email networks also appear at Fig. 6 and show the strongest relationship. The gradient, lighter to darker, indicates the increase in number of rewiring attempts. Increasing the assortativity through targeted rewiring increased the mean geodesic across all networks.

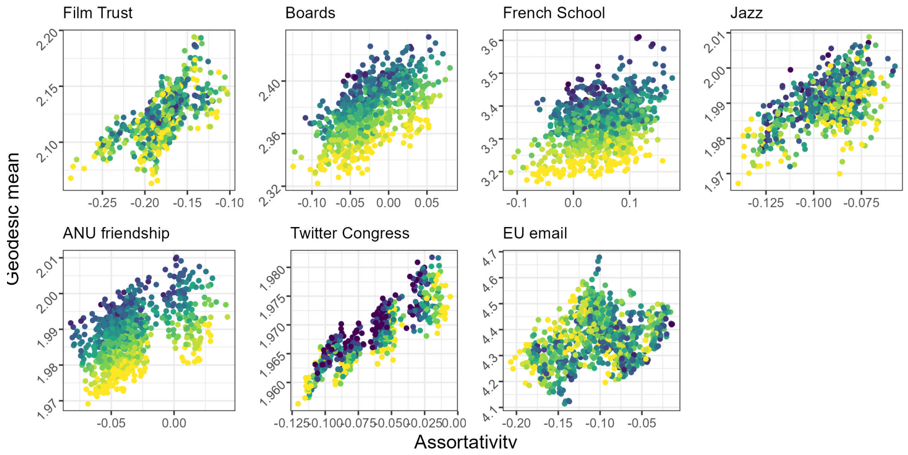

Figure 24: Assortativity and Geodesic mean pair combination with clustering coefficient rewiring. Consistent with the EU Email networks in Fig. 6, no pattern was observed across all networks. The gradient, lighter to darker, indicates the increase in number of rewiring attempts. We found no relationship between increasing the clustering coefficient through targeted rewiring and changes in the assortativity or mean geodesic for any of the networks.

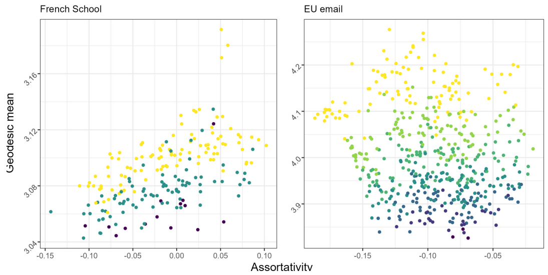

Figure 25: Assortativity and Geodesic mean pair combination with mean geodesic rewiring. Consistent with the EU Email networks in Fig. 6, no pattern was observed across all networks. The gradient, lighter to darker, indicates the increase in number of rewiring attempts. We found no relationship between decreasing the mean geodesic through targeted rewiring and changes in the assortativity for any of the networks.

Clustering coefficient and geodesic mean

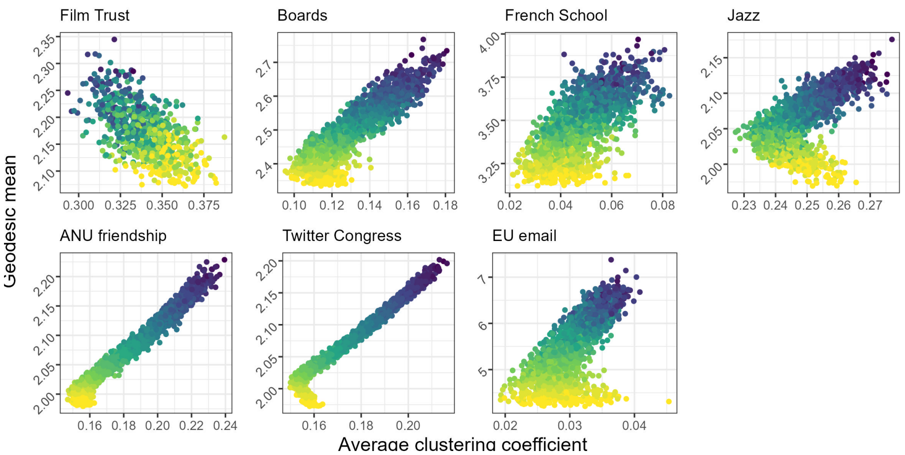

Figure 26: Clustering coefficient and Geodesic mean pair combination with assortativity rewiring. The Twitter Congress, also at Fig. 8, and Jazz network showed an inverse relationship at the start of the rewiring whereas the French school networks, also in Fig. 7, showed no relationship. The gradient, lighter to darker, indicates the increase in number of rewiring attempts. Increasing the assortativity through targeted rewiring, increased both the clustering coefficient and mean geodesic, except for the French primary school network. The Twitter Congress and Jazz networks had an initial inverse relationship for the lower values of clustering coefficient which was later reversed.

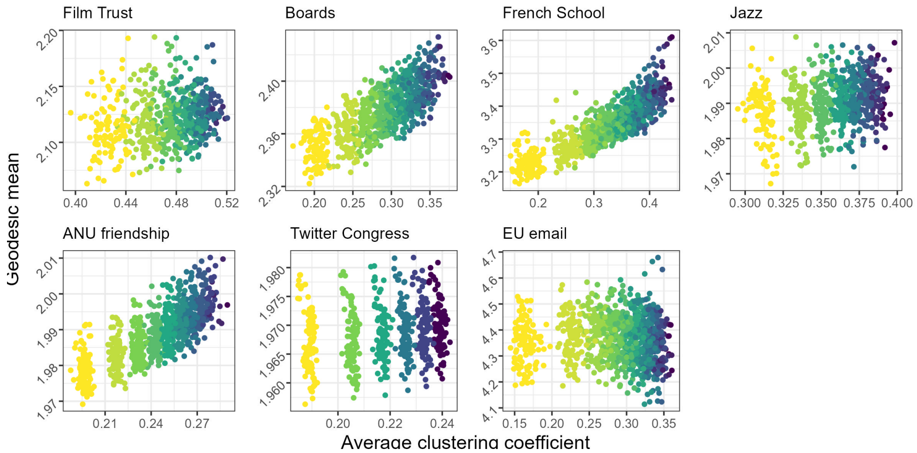

Figure 27: Clustering coefficient and Geodesic mean pair combination with clustering coefficient rewiring. The French school networks showed the strongest relationship and also appear at Fig. 7, whereas the Twitter Congress, also in Fig. 8, shows no pattern like the rest of the networks. The gradient, lighter to darker, indicates the increase in number of rewiring attempts. We found minimal or no relationship between increasing the clustering coefficient through targeted rewiring and changes in the mean geodesic for any of the networks.

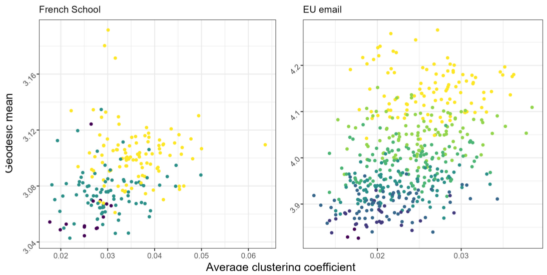

Figure 28: Clustering coefficient and Geodesic mean pair combination with mean geodesic rewiring. The French school networks also appear at Fig. 7, show the strongest relationship. The gradient, lighter to darker, indicates the increase in number of rewiring attempts. For the French primary school network we found a slight decrease in clustering coefficient as we decreased the mean geodesic through targeted rewiring. No other relationships were observed for the rest of the networks.

Local and global (transitivity) clustering coefficient

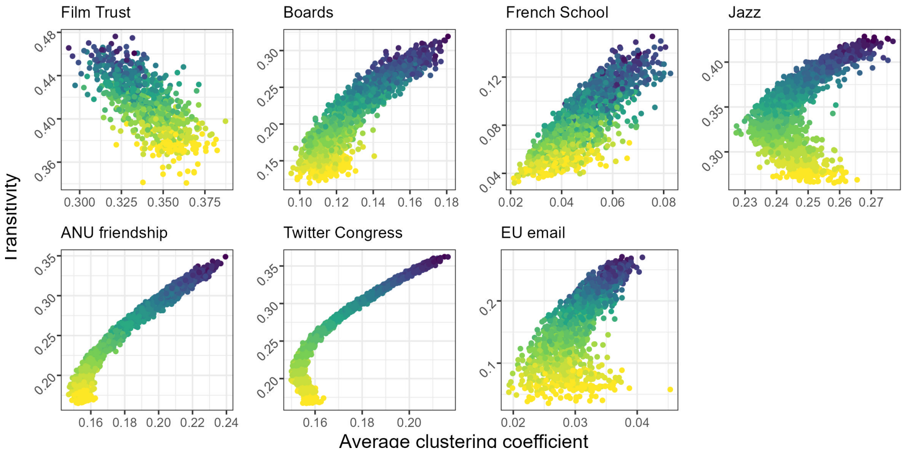

Figure 29: Local and global (transitivity) clustering coefficient pair combination with assortativity rewiring. A representative example of this relationship was the French school networks, also in Fig. 9, whereas the FilmTrust networks, also in Fig. 11, show the inverse relationship. The gradient, lighter to darker, indicates the increase in number of rewiring attempts. Increasing the assortativity through targeted rewiring, increased both the local and global clustering coefficient across all networks.

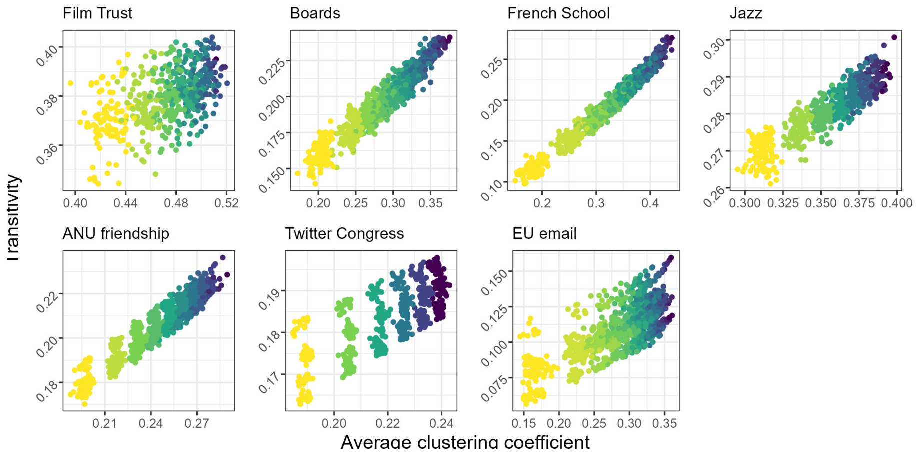

Figure 30: Local and global (transitivity) clustering coefficient Mean pair combination with clustering coefficient rewiring. A typical example was the French school networks, also in Fig. 9, consistent across all other networks. The gradient, lighter to darker, indicates the increase in number of rewiring attempts. Increasing the clustering coefficient through targeted rewiring, increased both the local and global clustering coefficient across all networks.

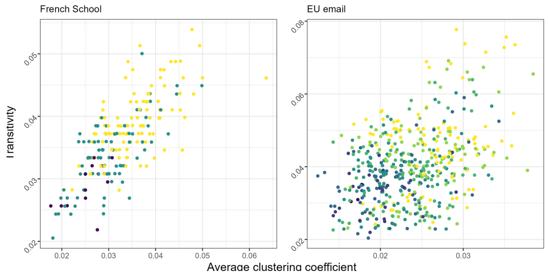

Figure 31: Local and global (transitivity) clustering coefficient pair combination with mean geodesic rewiring. The French school networks, also appear at Fig. 9, show the strongest relationship. The gradient, lighter to darker, indicates the increase in number of rewiring attempts. We found no relationship between decreasing the mean geodesic through targeted rewiring and changes in the local and global clustering coefficient for neither of the networks.

Mean closeness centrality

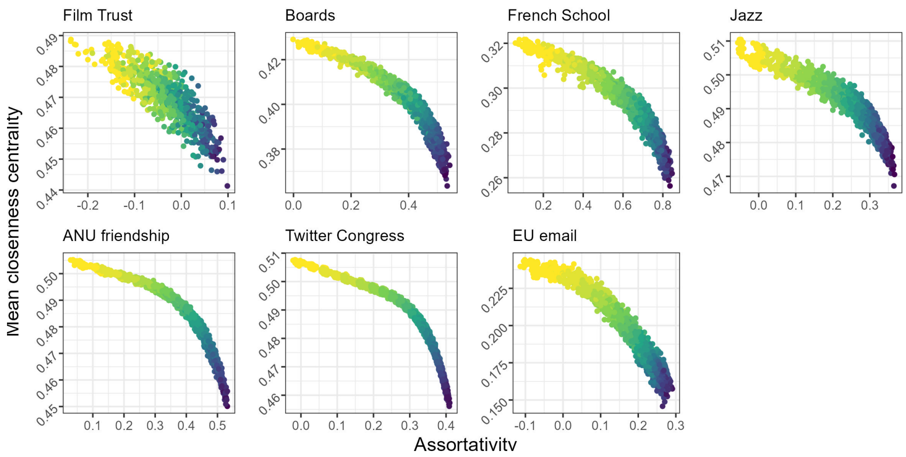

Figure 32: Mean closeness centrality and Assortativity pair combination with assortativity rewiring. The French school networks, also appear at Fig. 12, show the strongest relationship, consistent across networks. The gradient, lighter to darker, indicates the increase in number of rewiring attempts. Increasing the assortativity through targeted rewiring slightly decreased the mean closeness centrality but not the mean betweenness or eigenvector centralities across all networks.

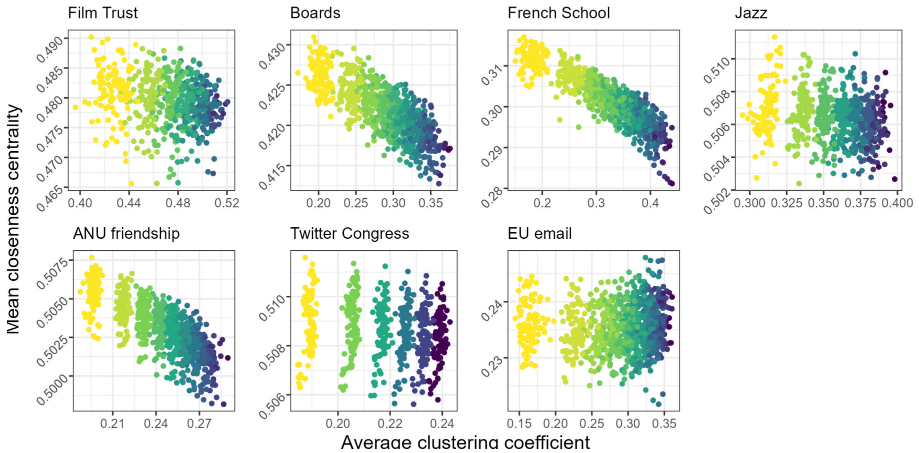

Figure 33: Mean closeness centrality and Clustering coefficient pair combination with clustering coefficient rewiring. The French School networks, also in Fig. 13, and Boards showed a positive relationship, not consistent across the other networks. The gradient, lighter to darker, indicates the increase in number of rewiring attempts. Increasing the clustering coefficient through targeted rewiring slightly decreased the mean closeness centrality but not the mean betweenness or eigenvector centralities across all networks.

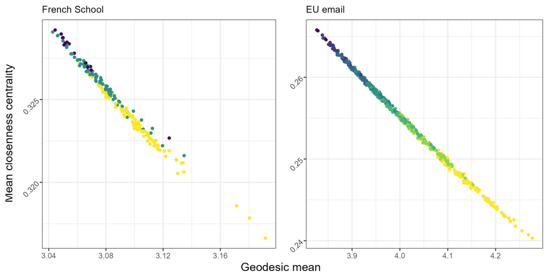

Figure 34: Mean closeness centrality and Mean Geodesic pair combination with mean geodesic rewiring. The EU email networks, also in Fig. 14, showed the strongest relationship. The gradient, lighter to darker, indicates the increase in number of rewiring attempts. Decreasing the mean geodesic through targeted rewiring slightly increased the mean closeness centrality but not the mean betweenness or eigenvector centralities across all networks.

Mean and Gini closeness centrality

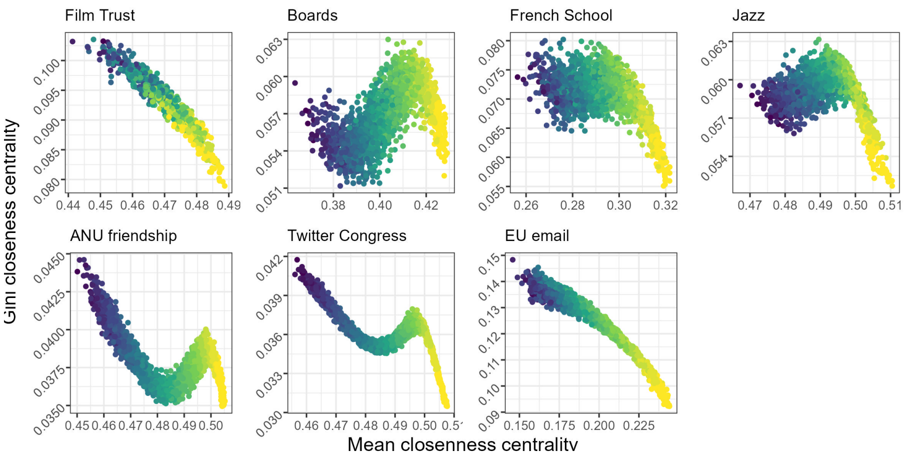

Figure 35: Mean and Gini closeness centrality pair combination with assortativity rewiring. The ANU friendship networks, also in Fig. 15, and Twitter Congress have a inverse relationship, unlike the rest of the networks. The gradient, lighter to darker, indicates the increase in number of rewiring attempts. Increasing the assortativity through targeted rewiring slightly decreased the mean closeness centrality and increased the Gini closeness centrality across all networks.

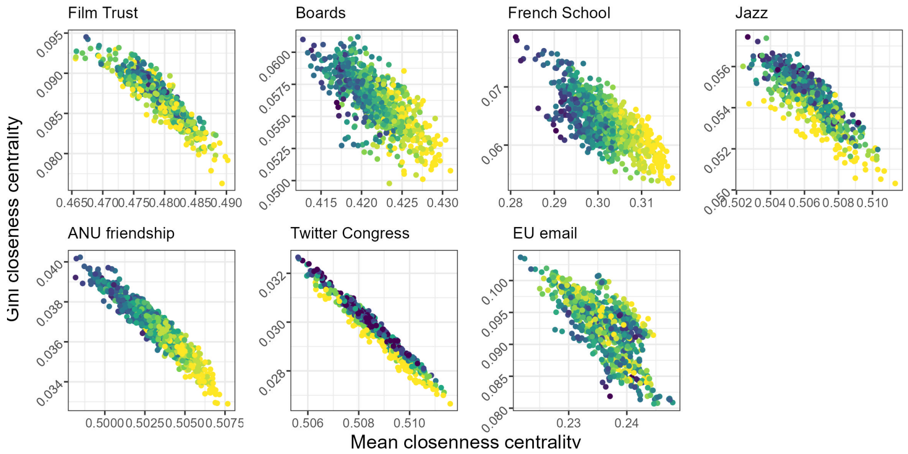

Figure 36: Mean and Gini closeness centrality pair combination with clustering coefficient rewiring. Consistent with ANU friendship networks, also in Fig. 15, no pattern was observed across all networks. The gradient, lighter to darker, indicates the increase in number of rewiring attempts. Increasing the clustering coefficient through targeted rewiring decreased the mean closeness centrality and increased the Gini closeness centrality across all networks.

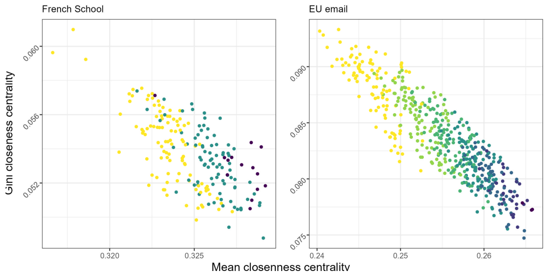

Figure 37: Mean and Gini closeness centrality pair combination with mean geodesic rewiring. The EU email networks, also in Fig. 14, showed the strongest relationship. The gradient, lighter to darker, indicates the increase in number of rewiring attempts. Decreasing the mean geodesic through targeted rewiring increased the mean closeness centrality and decreased the Gini closeness centrality but only for the EU Email network. Note that the changes are very small.

Mean betweenness centrality

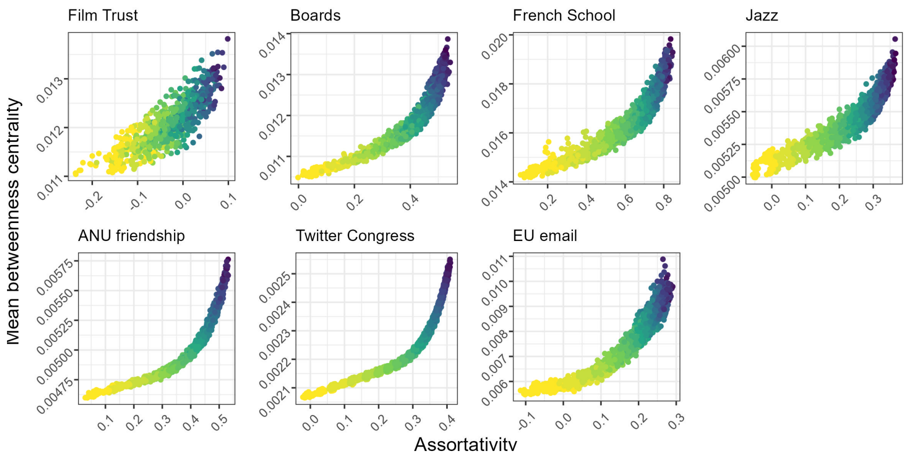

Figure 38: Mean betweenness centrality and Assortativity pair combination with assortativity rewiring. Consistent with French school networks, also in Fig. 12, there is no relationship between both properties. The gradient, lighter to darker, indicates the increase in number of rewiring attempts. Increasing the assortativity through targeted rewiring slightly increased the mean betweenness centrality across all networks. Note that the changes are very small.

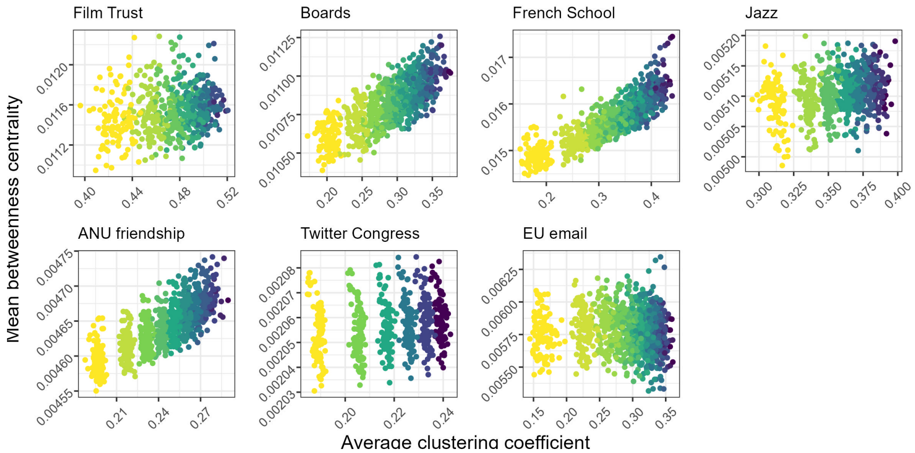

Figure 39: Mean betweenness centrality and Clustering coefficient pair combination with clustering coefficient rewiring. Consistent with French school networks, also in Fig. 13, there is no relationship between both properties. The gradient, lighter to darker, indicates the increase in number of rewiring attempts. Increasing the clustering coefficient through targeted rewiring slightly increased the mean betweenness centrality for some networks and showed no pattern for others. Note that the changes are very small.

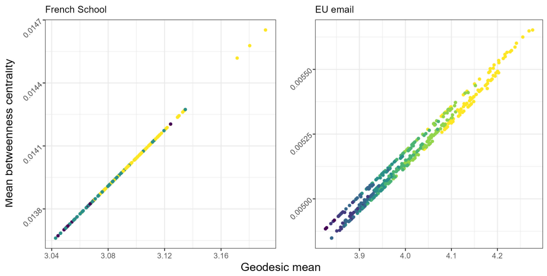

Figure 40: Mean betweenness centrality and Mean Geodesic pair combination with mean geodesic rewiring. Consistent with the EU email networks, also in Fig. 14, there is no relationship between both properties. The gradient, lighter to darker, indicates the increase in number of rewiring attempts. Decreasing the mean geodesic through targeted rewiring decreased the mean betweenness centrality across networks.

Mean and Gini betweenness centrality

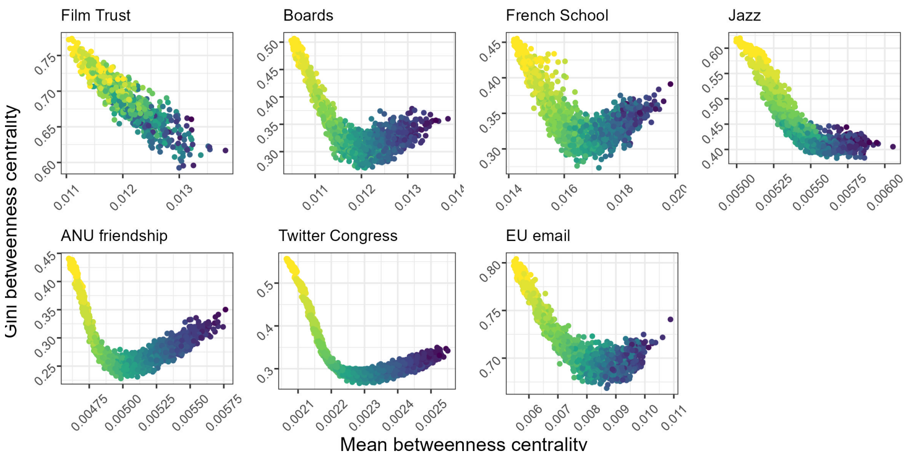

Figure 41: Mean and Gini betweenness centrality pair combination with assortativity rewiring. The FilmTrust project networks, also in Fig. 16, are a typical example of this inverse relationship. The gradient, lighter to darker, indicates the increase in number of rewiring attempts. Increasing the assortativity through targeted rewiring slightly decreased the mean betweenness centrality and increased the Gini betweenness centrality across networks.

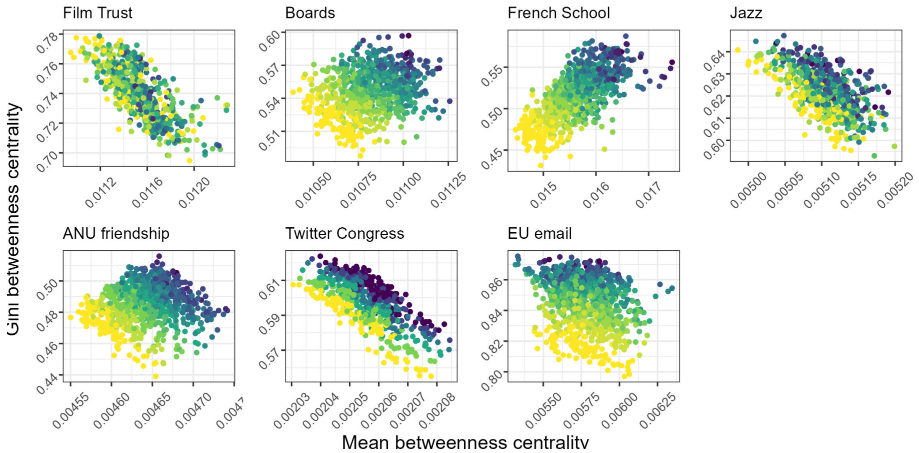

Figure 42: Mean and Gini betweenness centrality pair combination with clustering coefficient rewiring. Consistent with the FilmTrust project networks, also in Fig. 16, there is no relationship between both properties. The gradient, lighter to darker, indicates the increase in number of rewiring attempts. Increasing the clustering coefficient through targeted rewiring decreased the mean betweenness centrality and increased the Gini betweenness centrality.

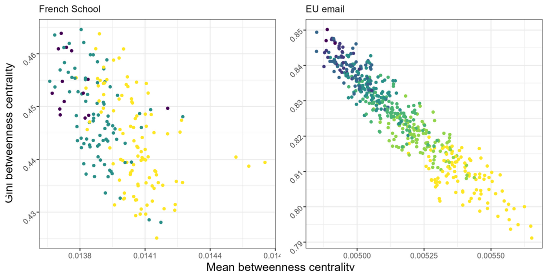

Figure 43: Mean and Gini betweenness centrality pair combination with mean geodesic rewiring. There is no relationship between both properties. The gradient, lighter to darker, indicates the increase in number of rewiring attempts. Decreasing the mean geodesic through targeted rewiring decreased the mean betweenness centrality and increased the Gini betweenness centrality but only for the EU Email network. Note that the changes are very small.

Mean Eigenvector Centrality

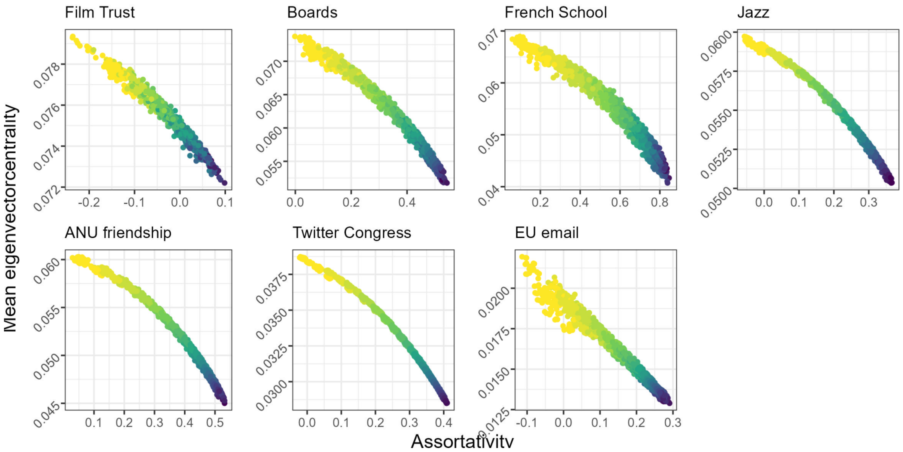

Figure 44: Mean Eigenvector Centrality and Assortativity pair combination with assortativity rewiring. The French school networks, also appear at Fig. 12, show the strongest relationship. The gradient, lighter to darker, indicates the increase in number of rewiring attempts. Increasing the assortativity through targeted rewiring slightly decreased the mean eigenvector centrality across all networks. Note that the changes are very small.

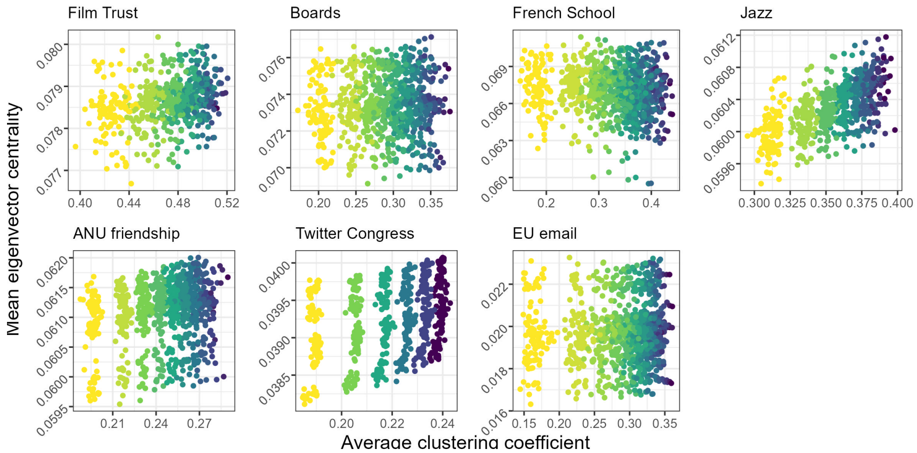

Figure 45: Mean Eigenvector Centrality and Clustering coefficient pair combination with clustering coefficient rewiring. The French school networks, also appear at Fig. 13, show no relationship between these properties. The gradient, lighter to darker, indicates the increase in number of rewiring attempts. Increasing the clustering coefficient through targeted rewiring showed no relationship with the mean eigenvector centrality across all networks.

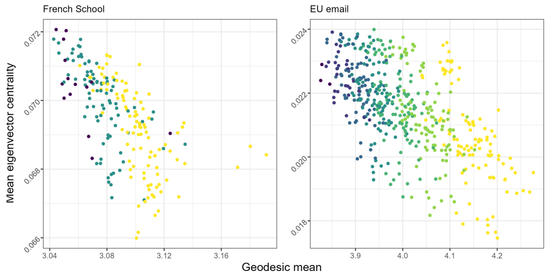

Figure 46: Mean Eigenvector Centrality and Mean Geodesic pair combination with mean geodesic rewiring. The EU email networks, also appear at Fig. 14 show no relationship between these properties. The gradient, lighter to darker, indicates the increase in number of rewiring attempts. Decreasing the mean geodesic through targeted rewiring showed a slight decrease in the mean eigenvector centrality but the pattern is unclear.
